# Platelet factors attenuate inflammation and rescue cognition in ageing

**DOI:** 10.1038/s41586-023-06436-3

**Published:** 2023-08-16

**Authors:** Adam B. Schroer, Patrick B. Ventura, Juliana Sucharov, Rhea Misra, M. K. Kirsten Chui, Gregor Bieri, Alana M. Horowitz, Lucas K. Smith, Katriel Encabo, Imelda Tenggara, Julien Couthouis, Joshua D. Gross, June M. Chan, Anthony Luke, Saul A. Villeda

**Affiliations:** 1 Department of Anatomy, University of California San Francisco, San Francisco, CA USA; 2 Biomedical Sciences Graduate Program, University of California San Francisco, San Francisco, CA USA; 3 Department of Urology, University of California San Francisco, San Francisco, CA USA; 4 Department of Genetics, Stanford University School of Medicine, Stanford, CA USA; 5 Department of Cell Biology, Duke University, Durham, NC USA; 6 Departments of Epidemiology and Biostatistics, University of California San Francisco, San Francisco, CA USA; 7 Department of Orthopaedics, University of California San Francisco, San Francisco, CA USA; 8 Department of Physical Therapy and Rehabilitation Science, University of California San Francisco, San Francisco, CA USA; 9 Bakar Aging Research Institute, University of California San Francisco, San Francisco, CA USA

**Keywords:** Neuroimmunology, Cognitive ageing

## Abstract

Identifying therapeutics to delay, and potentially reverse, age-related cognitive decline is critical in light of the increased incidence of dementia-related disorders forecasted in the growing older population^1^. Here we show that platelet factors transfer the benefits of young blood to the ageing brain. Systemic exposure of aged male mice to a fraction of blood plasma from young mice containing platelets decreased neuroinflammation in the hippocampus at the transcriptional and cellular level and ameliorated hippocampal-dependent cognitive impairments. Circulating levels of the platelet-derived chemokine platelet factor 4 (PF4) (also known as CXCL4) were elevated in blood plasma preparations of young mice and humans relative to older individuals. Systemic administration of exogenous PF4 attenuated age-related hippocampal neuroinflammation, elicited synaptic-plasticity-related molecular changes and improved cognition in aged mice. We implicate decreased levels of circulating pro-ageing immune factors and restoration of the ageing peripheral immune system in the beneficial effects of systemic PF4 on the aged brain. Mechanistically, we identified CXCR3 as a chemokine receptor that, in part, mediates the cellular, molecular and cognitive benefits of systemic PF4 on the aged brain. Together, our data identify platelet-derived factors as potential therapeutic targets to abate inflammation and rescue cognition in old age.

## Abstract (web summary)

Platelet factors transfer the benefits of young blood to the ageing brain in mice through CXCR3, which mediates the cellular, molecular and cognitive benefits of systemic PF4 on the aged brain.

## Main

Systemic rejuvenating interventions—such as heterochronic parabiosis (in which the circulatory systems of young and aged animals are joined)—can reverse age-related impairments in neurogenesis, synaptic plasticity and cognitive function in aged mice^2–7^. Although the field of rejuvenation research is fast growing, the underlying components in the blood of young animals responsible for reversing age-related brain impairments remain largely unclear. Reports using heterochronic parabiosis led to studies in which systemic administration of blood plasma preparations derived from young or exercised mice was demonstrated to rejuvenate the aged brain. These plasma preparations are in large part devoid of cellular components; however, the universally used approach to generate plasma from the blood of young mice in these studies yields plasma preparations that contain both soluble factors and platelets^3,4,6–10^. We therefore sought to define the cellular and molecular mechanisms in the blood of young animals that drive the beneficial effects of systemically administering young blood plasma on the aged brain.

## Platelet factors abate neuroinflammation

**Fig. 1:**
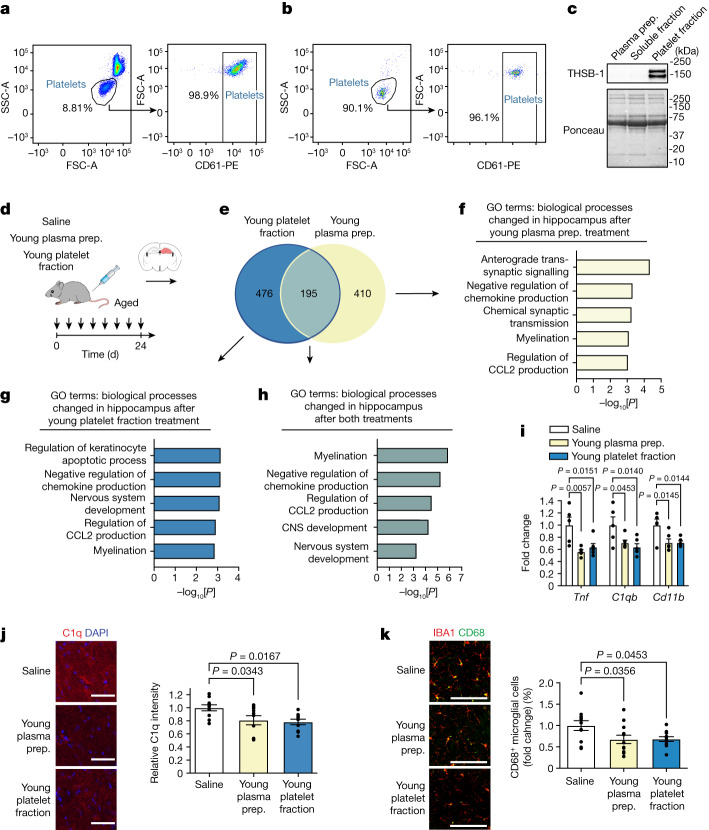
Platelet factors mitigate neuroinflammation in the aged hippocampus. **a**,**b**, Flow cytometry analysis of CD61^+^ platelets in mouse whole blood (**a**) and in the platelet fraction of young plasma preparations (**b**). **c**, Western blot of platelet marker thrombospondin-1 (THSB-1) in the young plasma preparation (prep.), the platelet-depleted fraction and the platelet fraction of mice. **d**, The timeline of administration of each treatment to aged (20 months) male mice. **e**–**h**, RNA-seq analysis of the hippocampus of aged mice after systemic treatment with young plasma preparation (yellow; *n* = 6 mice) or young platelet fraction (blue; *n* = 6 mice) relative to aged saline-treated mice (*n* = 5 mice). **e**, DEGs (*P* < 0.05) in the hippocampus of aged mice. **f**–**h**, GO terms associated with DEGs after treatment with young plasma preparation (**f**) and young platelet fraction (**g**) and the overlapping DEGs (**h**). **i**, qPCR analysis of neuroinflammation-related gene expression relative to *Gapdh* in the hippocampus of aged mice. *n* = 5 (saline), 6 (young plasma) and 6 (young platelet fraction) mice. **j**,**k**, Representative images and quantification of the C1q signal intensity (**j**; *n* = 10 mice per group) and IBA1^+^ and CD68^+^ cells (**k**; *n* = 11 (saline), 12 (young plasma) and 11 (platelet fraction) mice) in the dentate gyrus of the aged hippocampus. Uncropped immunoblots are provided in Supplementary Fig. 1. Scale bars, 25 μm (**j**) and 100 μm (**k**). Data are mean ± s.e.m. Statistical analysis was performed using Fisher’s exact tests (**f**–**h**) and one-way analysis of variance (ANOVA) with Dunnett’s post hoc test (**i**–**k**). Source data

To functionally investigate cellular components remaining in the blood plasma preparations of young mice (hereafter, the young blood plasma preparation), we used a centrifugation-based approach to collect the platelet fraction of young mice (hereafter, the young platelet fraction) and examined the potential beneficial effects of its systemic administration on the aged brain. As a control, we confirmed platelet enrichment within the young platelet fraction (Fig. 1a–c). Aged male mice were subsequently intravenously injected with either the young blood plasma preparation, the young platelet fraction or saline (100 μl per injection) 8 times over 24 days (Fig. 1d). To investigate the molecular changes elicited in the aged brain by systemic administration of the young blood plasma preparation and the young platelet fraction, we performed RNA-sequencing (RNA-seq) analysis of the aged hippocampus—a brain region that is sensitive to the detrimental effects of ageing^11^. Relative to saline-treated aged control mice, administration of the young blood plasma preparation and the young platelet fraction resulted in the differential expression of 605 and 671 genes, respectively (Fig. 1e). Gene Ontology (GO) analysis of the overlapping 195 differentially expressed genes (DEGs) across the young blood plasma preparation and young platelet fraction treatments identified changes associated with immune regulation and nervous system development (Fig. 1f–h and Supplementary Table 1).

**Extended Data Fig. 1:**
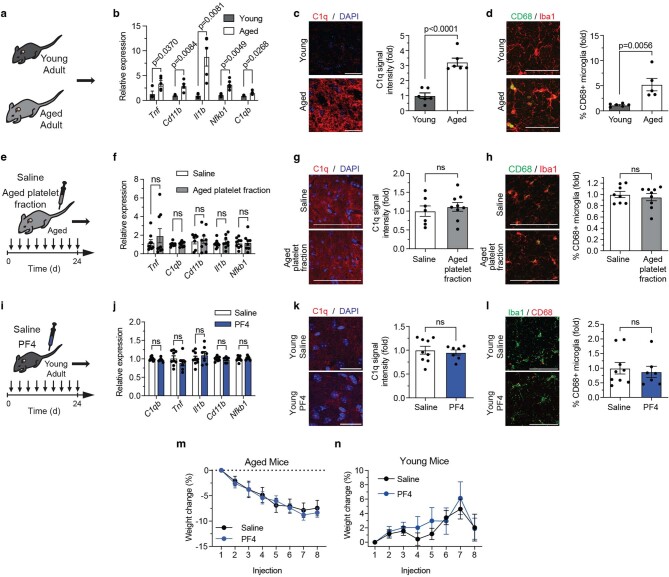
The beneficial effect of systemic administration of platelet factors on neuroinflammation in the hippocampus is age-dependent. (**a**) Schematic illustrates young (5 months) and aged mice (22 months) used for analyses. (**b**) qPCR of neuroinflammation-related gene expression relative to *Gapdh* in hippocampi from young and aged mice (n = 4 young; 5 aged mice). (**c,d**) Representative images and quantification of C1q signal intensity (**c**; n = 6 mice/group; scale bars = 25 µm) and Iba1- and CD68-positive cells (**d**; n = 6 young; 5 aged mice; scale bars = 50 µm) in the dentate gyrus of the hippocampus. (**e**) Schematic illustrates timeline of tail vein injection of saline or the aged platelet fraction of plasma into aged mice. (**f**) qPCR of neuroinflammation-related gene expression relative to *Gapdh* in aged hippocampi (n = 9 mice/group). (**g,h**) Representative images and quantification of C1q signal intensity (**g**; n = 7 saline; 9 aged platelet fraction mice; scale bars = 50 µm) and Iba1- and CD68-positive cells (**h**; n = 8 saline; 9 aged platelet fraction mice; scale bars = 50 µm) in the dentate gyrus of the aged hippocampus. (**i**) Schematic illustrates timeline of saline or PF4 administration to young mice (3 months). (**j**) qPCR of neuroinflammation-related gene expression relative to *Gapdh* in young hippocampi (n = 9 saline; 7 PF4 mice). (**k,l**) Representative images and quantification of C1q signal intensity (**k**; n = 9 saline; 7 PF4 mice; scale bars = 10 µm) and Iba1- and CD68-positive cells (**l**; n = 9 saline; 7 PF4 mice; scale bars = 50 µm) in the dentate gyrus of the young hippocampus. (**m–n**) Percent change in weight of aged (**m**; n = 16 saline; 12 PF4 mice) and young (**n**; n = 9 saline; 8 PF4 mice) mice during treatment. Data shown as mean±s.e.m.; two-tailed unpaired *t*-test (**b-d,f-h,j-l**), two-way ANOVA with Šidák’s post-hoc test (**m,n**). Source data

Maladaptive inflammation is a hallmark of brain ageing^12^—with increased expression of pro-inflammatory genes, complement cascade initiation and microglial activation (Extended Data Fig. 1a–d). Correspondingly, we examined these neuroinflammation markers in an independent cohort of aged male mice after systemic administration of the young blood plasma preparation, the young platelet fraction or saline. We detected a decrease in the expression of the pro-inflammatory cytokine tumour necrosis factor (*Tnf*), complement initiator *C1qb* and microglia activation marker *CD11b* on the basis of quantitative PCR (qPCR) analysis of the hippocampus of aged mice after systemic administration of the young blood plasma preparation or the young platelet fraction compared with saline (Fig. 1i); we likewise detected a decrease in the level of C1q protein in the aged hippocampus as determined by immunohistochemical analysis (Fig. 1j). We analysed microglia, the resident macrophages of the brain, and observed a decrease in the levels of IBA1-positive microglia co-expressing the lysosomal activation marker CD68 in the hippocampus of aged mice after systemic administration with the young blood plasma preparation or the young platelet fraction (Fig. 1k). No differences in neuroinflammation markers were observed in aged male mice after systemic administration of the aged platelet fraction compared with saline (Extended Data Fig. 1e–h). Our data indicate that systemic administration of the young plasma preparation decreases neuroinflammation in the aged hippocampus, and that these benefits are transferred, at least in part, through factors within the young platelet fraction. These findings also demonstrate that the benefits of the young blood plasma preparation on the aged brain extend beyond previously reported rejuvenating effects on adult neurogenesis and synaptic plasticity^2–7^.

## PF4 is elevated in young blood plasma

**Fig. 2:**
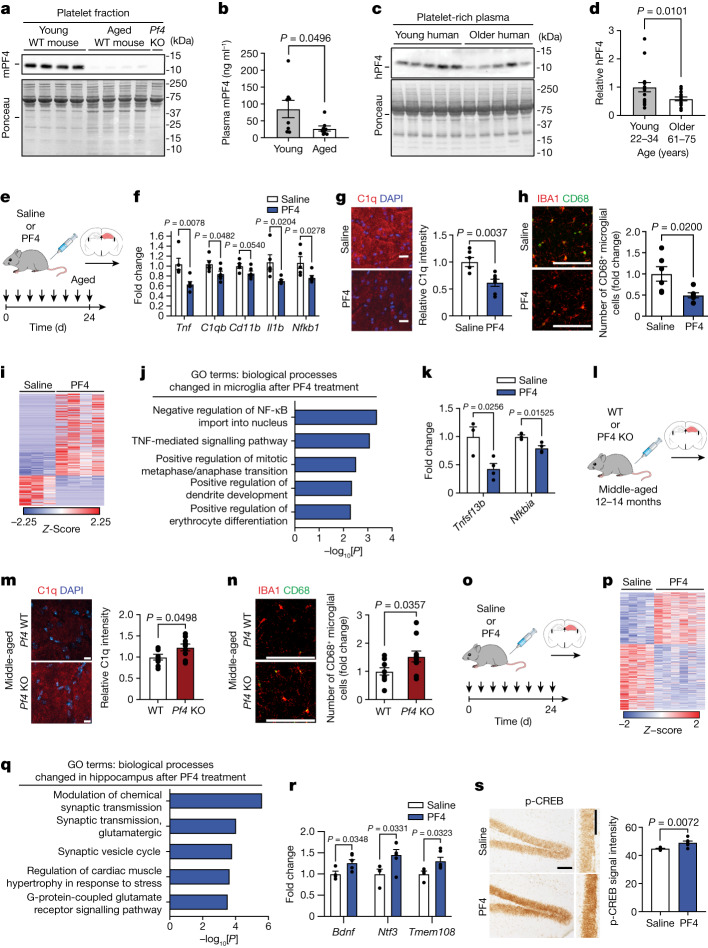
Systemic PF4 attenuates neuroinflammation and elicits synaptic-related changes in the aged hippocampus. **a**,**b**, Western blot analysis of PF4 in the platelet fraction (*n* = 4 independent samples per group) (**a**) and ELISA of PF4 in blood plasma preparations from young (3 months) and aged (20 months) mice (*n* = 8 mice per group) (**b**). **c**,**d**, Western blot analysis (**c**) and quantification (**d**) of PF4 in platelet-rich plasma from young (27.1 ± 3.7 years) and older (66.3 ± 4.2 years; *n* = 16 participants per group) humans. **e**, The timeline of saline or PF4 administration to aged male mice. **f**, qPCR analysis of neuroinflammation-related gene expression relative to *Gapdh* in the aged hippocampus (*n* = 5 saline; 6 PF4 mice). **g**,**h**, Representative images and quantification of C1q signal intensity (**g**; *n* = 6 (saline) and 7 (PF4) mice) and IBA1^+^ and CD68^+^ cells (**h**; *n* = 6 mice per group) in the dentate gyrus of the aged hippocampus. **i**–**k**, Significant DEGs (*P* < 0.01) after RNA-seq analysis (**i**), associated GO terms (**j**) and the fold change in expression (fragments per kilobase of transcript per million mapped reads (FPKM)) of TNF-signalling-related genes (**k**) in aged hippocampal microglia (*n* = 3 (saline) and 4 (PF4) mice). **l**, Schematic of middle-aged (12–14 months) WT and *Pf4*-deficient (*Pf4* KO) male mice. **m**,**n**, Representative images and quantification of C1q signal intensity (**m**; *n* = 8 WT; 9 *Pf4*-KO mice) and IBA1^+^ and CD68^+^ cells (**n**; *n* = 10 mice per group) in the dentate gyrus of the middle-aged hippocampus. **o**, The timeline of saline or PF4 administration to aged male mice. **p**–**r**, Significant DEGs (*P* < 0.05) after RNA-seq analysis (**p**), associated GO terms (**q**) and the fold change in expression (FPKM) of synaptic-transmission-related genes (**r**) in the aged hippocampus (*n* = 4 (saline) and 6 (PF4) mice). **s**, Representative images and quantification of CREB phosphorylation (p-CREB) immunolabelling in the aged hippocampus (*n* = 6 mice per group). Uncropped immunoblots are provided in Supplementary Fig. 1. Data are mean ± s.e.m. Statistical analysis was performed using two-tailed unpaired *t*-tests (**b**, **d**, **f**–**h**, **k**, **m**, **n**, **r** and **s**) and Fisher’s exact tests (**j** and **q**). Scale bars, 10 μm (**g** and **m**), 100 μm (**h** and **n**) and 150 μm (**s**). Source data

We next sought to gain mechanistic insights into individual circulating factors mediating the observed beneficial effects of systemically administering the young platelet fraction. PF4—a chemokine released from platelets and involved in coagulation and immunomodulatory functions^13,14^—is elevated in the blood serum after neutral blood exchange in aged mice (a systemic rejuvenating intervention^15^) as well as therapeutic plasma exchange in humans (a similar human clinical procedure^16^). Moreover, PF4 has been implicated in the beneficial effects of exercise on neurogenesis in the young brain^17^. Although inconsistent evidence for age-related bidirectional changes in the levels of platelet-derived PF4 exist in the literature, reports have shown decreased PF4 in the plasma of humans and non-human primates with age^18^. We detected higher PF4 levels in the platelet fraction of young mice compared with aged mice by western blot analysis (Fig. 2a), and elevated levels were detected in the blood plasma preparations from young mice on the basis of an enzyme-linked immunosorbent assay (ELISA) (Fig. 2b). Moreover, we detected elevated levels of PF4 in platelet-rich plasma derived from the blood of young compared with older healthy human individuals by western blot analysis (Fig. 2c,d). Correspondingly, we elected to focus our functional studies on PF4. Note that the perceived discrepancy in PF4 levels observed with age across groups may be the result of methodological differences. Whereas blood centrifuged at high speeds generates platelet-poor plasma, blood plasma preparations used to promote hippocampal rejuvenation contain remaining platelets that could differ in platelet number and activation state, consequently influencing the levels of PF4 detected with age.

## PF4 reduces neuroinflammation

To investigate the potential pro-youthful activity of PF4, aged male mice were systemically administered with carrier-free recombinant mouse PF4 (5 μg ml^−1^) or saline 8 times over 24 days (100 μl per injection), and neuroinflammation was assessed in the hippocampus of aged mice (Fig. 2e). We detected decreased hippocampal expression of pro-inflammatory genes *Tnf*, *Nfkb1* and *Il1b*, complement factor *C1qb* and microglia activation marker *CD11b* in aged mice that were administered with PF4 compared with in those administered with saline, as determined using qPCR (Fig. 2f). Furthermore, we observed reduced C1q levels and a decrease in activated CD68^+^ microglia in the hippocampus of aged mice after PF4 treatment on the basis of immunohistochemistry analysis (Fig. 2g,h). To further investigate microglia-specific molecular changes, we performed RNA-seq analysis of microglia isolated from hippocampal tissue of aged mice after intravenous injections with PF4 or saline and found 346 DEGs (Fig. 2i and Supplementary Table 2). GO analysis identified changes in TNF-mediated signalling (Fig. 2j), a pro-inflammatory signalling cascade previously linked to impaired synaptic strength and cognitive dysfunction in neurodegenerative disease^19,20^. PF4 treatment also decreased expression of inflammatory signals, such as *Nfkbia* and *Tnfsf13b* (Fig. 2k). No differences in neuroinflammation markers were observed in young male mice administered with PF4 compared with saline (Extended Data Fig. 1i–l), indicating an age-dependent effect of PF4 on the hippocampus. None of the mice showed adverse effects or differences in weight change regardless of treatment (Extended Data Fig. 1m,n).

Finally, we investigated whether the loss of PF4 induced neuroinflammation in the adult hippocampus (Fig. 2l). We observed an increase in C1q levels and CD68^+^ microglia in the hippocampus of middle-aged *Pf4* knockout (*Pf4-*KO) mice compared with their littermate wild-type (WT) control mice (Fig. 2m,n). Together, these cellular and transcriptomics data indicate that systemic administration of PF4 is sufficient to attenuate neuroinflammation in the aged hippocampus, whereas the loss of PF4 accelerates increased neuroinflammation by middle age.

## PF4 elicits synaptic-related changes

To further investigate the molecular changes elicited by systemic PF4 treatment more broadly, we performed RNA-seq analysis of the hippocampus of aged mice after intravenous injections with PF4 or saline (Fig. 2o,p and Supplementary Table 3). GO analysis of DEGs identified changes associated with synaptic transmission (Fig. 2q). PF4 treatment also increased expression of synaptic-plasticity-related markers, such as *Bdnf*, *Ntf3* and *Tmem108* (Fig. 2r). Previously, we demonstrated that activation of the cAMP response element binding protein (CREB)—through phosphorylation at Ser133—in the aged hippocampus, in part, mediates synaptic-plasticity-related enhancements induced by systemic exposure to young blood^3^. Similarly, we observed an increase in the levels of CREB phosphorylation in the hippocampus of aged mice that were systemically administered with PF4 compared with saline, as determined using immunohistochemistry (Fig. 2s). These transcriptomics data indicate that PF4 treatment elicits synaptic-plasticity-related molecular changes in the aged hippocampus.

## PF4 rejuvenates the ageing immune system

**Fig. 3:**
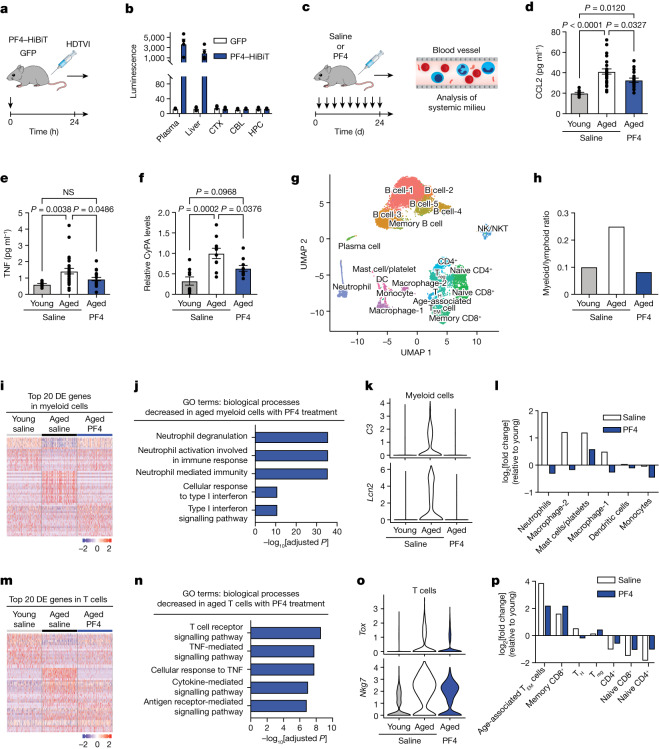
Systemic PF4 restores the ageing peripheral immune system to a more youthful state. **a**, The timeline of HDTVI of expression constructs to aged (20 months) male mice. **b**, Luminescence-based quantification of PF4–HiBiT in aged mice after HDTVI (*n* = 3 (GFP) and 4 (PF4–HiBiT) mice). **c**, The timeline of saline or PF4 administration to young (3 months) and aged male mice. **d**–**f**, Quantification of plasma levels of CCL2 (**d**; *n* = 9 (young, saline), 25 (aged, saline) and 17 (aged, PF4) mice) and TNF (**e**; *n* = 11 (young, saline), 27 (aged, saline) and 19 (aged, PF4) mice) by ELISA and CyPA (**f**; *n* = 9 (young, saline), 10 (aged, saline) and 10 (aged, PF4) mice) by western blotting. **g**–**p**, CITE-seq analysis of splenocytes from young and aged saline-treated controls, and aged PF4-treated mice (*n* = 5 pooled mice per group). **g**, Combined two-dimensional visualization of single-cell clusters. DC, dendritic cell; NK, natural killer cells; NKT, natural killer T cells; T_EM_, T effector memory cells; T_H_, T helper cells; T_reg_, regulatory T cells. **h**, Comparison of the ratio of myeloid cells to lymphoid cells in the spleen. **i**, The top 20 DEGs in myeloid cells from each group. **j**, GO terms associated with downregulated genes in aged myeloid cells after PF4 administration relative to the control. **k**, Complement *C3* and *Lcn2* expression in myeloid cells. **l**, The fold change in myeloid cell populations relative to young control mice. **m**, The top 20 DEGs in T cells from each group. **n**, GO terms associated with downregulated genes in aged T cells after PF4 administration relative to the control. **o**, *Tox* and *Nkg7* expression in T cells. **p**, The fold change in aged T cell populations relative to young control mice. Data are mean ± s.e.m., except for the violin plots in **k** and **o**. Statistical analysis was performed using one-way ANOVA with Tukey’s post hoc test (**d**–**f**) and Fisher’s exact test with false-discovery rate correction (**j** and **n**). Source data

**Extended Data Fig. 2:**
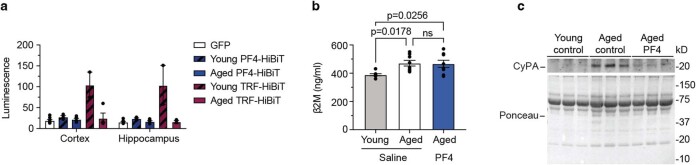
Systemic administration of PF4 does not cross the BBB in aged or young mice, but affects the systemic milieu. (**a**) Luminescence-based quantification of PF4-HiBiT and TRF-HiBiT in tissues from young (3 months) and aged (20 months) mice following HDTVI (n = 7 GFP; 3 young PF4-HiBiT; 6 aged PF4-HiBiT; 2 young TRF-HiBiT; 4 aged TRF-HiBiT mice). (**b**) Quantification of plasma levels of β2M by ELISA (n = 8 mice/group). (**c**) Representative Western blot of Cyclophilin A (CyPA) in blood plasma preparation. For uncropped immunoblots, see Supplementary Fig. 1. Data shown as mean±s.e.m.; one-way ANOVA with Tukey’s post-hoc test (**b**). Source data

To delineate potential central versus peripheral mechanisms of action of PF4, we evaluated the ability of systemic PF4 to cross the blood–brain barrier. We generated expression constructs encoding a HiBiT-tagged version of PF4. HiBiT is a small peptide that forms a complex with LgBiT to produce a luminescent signal^21^, enabling the sensitive detection of tagged proteins^7,21^. Aged mice were given hydrodynamic tail vein injections (HDTVI) with expression constructs encoding PF4–HiBiT or GFP, and the HiBiT levels were characterized across various tissues (Fig. 3a,b). Luminescent signal was detected in plasma and liver; however, no signal was detected in the brain above those observed in GFP-injected aged animals (Fig. 3b). In an independent cohort, young and aged mice were given HDTVI with expression constructs encoding PF4–HiBiT, transferrin (TRF)–HiBiT or GFP. As a control, we assessed the age-dependent decrease in TRF transport into the brain through receptor-mediated transcytosis^22^ and concordantly detected a luminescent signal in the brain of the young but not aged TRF–HiBiT-injected animals (Extended Data Fig. 2a). No signal was detected in the brain of either young or aged PF4–HiBiT-injected animals. Although we do not exclude a direct role for PF4 in the brain, these data suggest a peripheral mechanism of action.

We and others have previously demonstrated that the aged systemic milieu promotes hippocampal ageing^23,24^, in part, through increased systemic levels of pro-ageing immune factors such as CCL2, CyPA (also known as PPIA), TNF and β_2_-microglobulin^19,24–26^. Indeed, strategies altering systemic pro-youthful or pro-ageing factors have been proposed as approaches for brain rejuvenation. We therefore assessed whether PF4 administration reduced levels of known pro-ageing immune factors in blood plasma preparations (Fig. 3c), and observed a decrease in circulating CCL2, CyPA and TNF, but not β_2_-microglobulin, by ELISA and western blot analysis (Fig. 3d–f and Extended Data Fig. 2b,c). Notably, neutralization of circulating CyPA in the blood improves the cognitive function in aged mice^24^. These data posit a decrease in age-related systemic inflammatory signals as a potential mediator of the beneficial effects of PF4 on neuroinflammation, with relevance to cognitive function.

**Extended Data Fig. 3:**
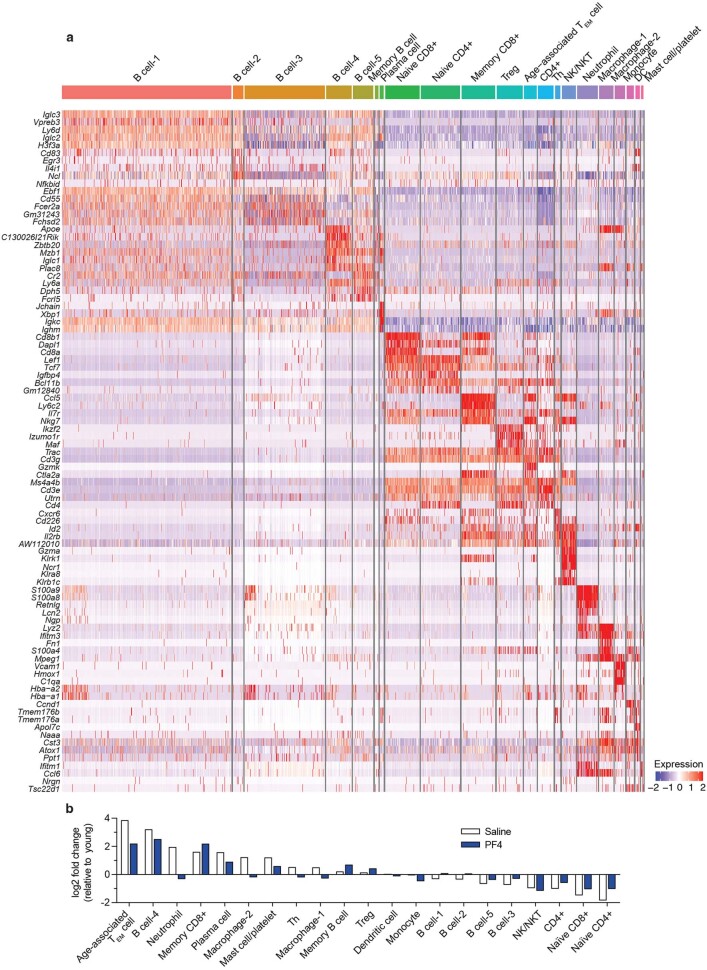
CITE-seq gene signature and populations for each cell cluster. (**a**) Heatmap of the expression levels of the top five marker genes for each of the 21 identified cell clusters. (**b**) Fold-change in cell populations from the spleen of aged saline treated control and aged PF4 treated mice, relative to young saline treated control mice (n = 5 pooled mice/group). Source data

Building on these findings, we next examined the effect of systemic PF4 administration more broadly on the aged peripheral immune system. We performed cellular indexing of transcriptomes and epitopes by sequencing (CITE-seq) analysis of splenocytes of aged male mice after intravenous injections with PF4 or saline, as well as of young saline-treated mice (Fig. 3c). Before single-cell gene expression analysis, cells were labelled with a panel of 34 antibodies for canonical surface markers (Extended Data Table 1). In total, 21 cell clusters were identified using a principal component analysis (PCA)-based approach and projected by uniform manifold approximation and projection (UMAP) onto a two-dimensional plot (Fig. 3g). Signatures for each cluster were generated by differential gene expression analyses to compare clusters (Extended Data Fig. 3a). Cell types were established on the basis of transcriptomic signature and canonical surface markers, and populations were compared across age and treatment groups (Extended Data Fig. 3b).

**Extended Data Fig. 4:**
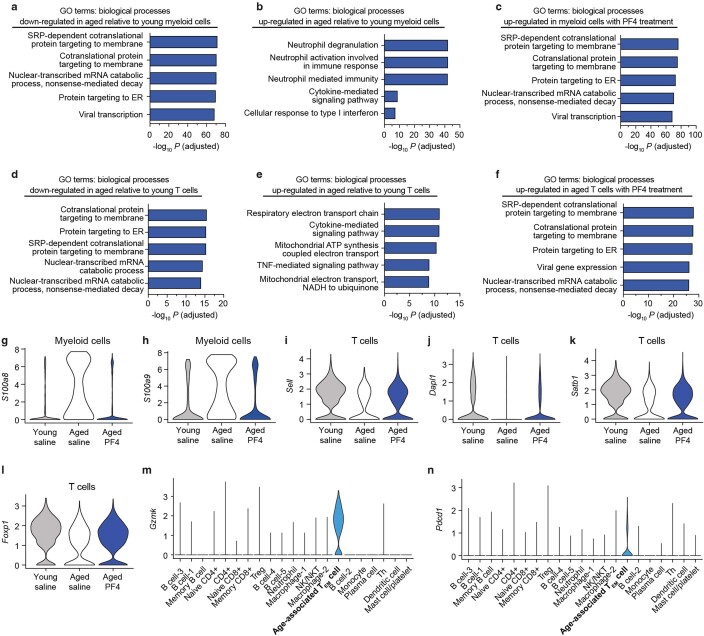
Systemic administration of PF4 restores spleen-derived immune cells to a more youthful state in aged mice. (**a**) Gene ontology (GO) terms associated with downregulated genes in myeloid cells from aged saline-treated mice relative to young saline-treated mice. (**b**) GO terms associated with upregulated genes in myeloid cells from aged saline-treated mice relative to young saline-treated mice. (**c**) GO terms associated with upregulated genes in myeloid cells following PF4 administration relative to aged saline-treated mice. (**d**) GO terms associated with downregulated genes in T cells from aged saline-treated mice relative to young saline-treated mice. (**e**) GO terms associated with upregulated genes in T cells from aged saline-treated mice relative to young saline-treated mice. (**f**) GO terms associated with upregulated genes in T cells following PF4 administration relative to aged saline-treated mice. (**g–h**) Violin plot of the expression levels in myeloid cells of inflammatory mediator genes (**g**) *S100a8* and (**h**) *S100a9* (n = 5 pooled mice/group). (**i–l**) Violin plot comparing the expression levels in T cells of genes associated with a naïve phenotype including (**i**) *Sell*, (**j**) *Dapl1*, (**k**) *Satb1*, and (**l**) *Foxp1* (n = 5 pooled mice/group). (**m-n**) Violin plots showing expression levels of (**m**) *Gzmk* and (**n**) *Pdcd1* (encoding PD-1) in all cell clusters (splenoctyes from 3 groups with 5 pooled mice/group). Fisher exact test with False Discovery Rate correction (**a–f**).

**Extended Data Fig. 5:**
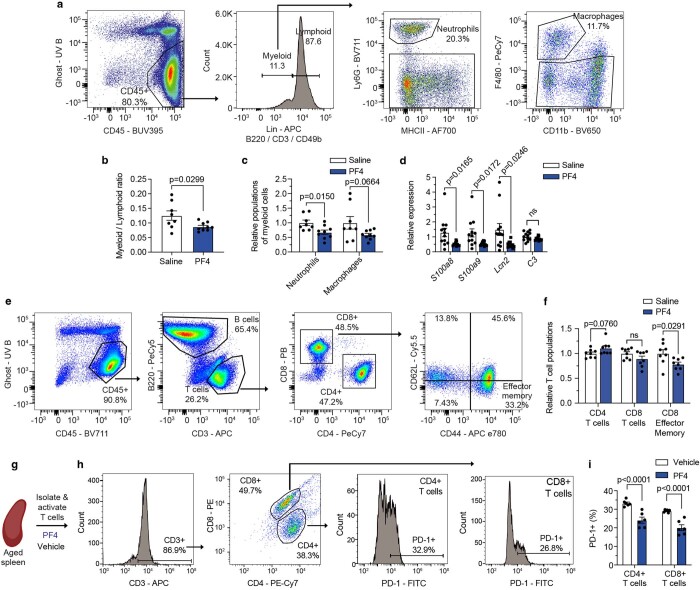
Validation of PF4-mediated changes to spleen-derived immune cells in aged mice. (**a-c**) Flow cytometry gating strategy for the identification of CD45+ myeloid (lin−) and lymphoid (lin+) cells, neutrophils (CD45+, lin−, Ly6G+), and macrophages (CD45+, lin−, F4/80+, CD11b+) in the aged spleen. (**b**) Comparison of the ratio of myeloid cells to lymphoid cells in the spleen of saline and PF4 treated aged mice (n = 8 saline; 9 PF4 mice). (**c**) Comparison of the relative populations of neutrophils and macrophages in the spleen (n = 8 saline; 9 PF4 mice). (**d**) qPCR of inflammation-related genes relative to *Gapdh* in the aged spleen (n = 12 saline; 11 PF4 mice). (**e–f**) Flow cytometry gating strategy for the identification of CD4+ T cells, CD8+ T cells, and CD8+ T effector memory cells (CD45+, CD3+, CD8+, CD44^HI^, CD62L^LO^) in the aged spleen. (**f**) Comparison of the relative populations of T cells in the spleen (n = 8 mice/group). (**g**) Schematic of aged T cell isolation, activation, and treatment with vehicle or PF4 (1 μg ml^−1^). (**h,i**) Flow cytometry gating strategy for the identification of the exhaustion marker PD1 in CD4+ and CD8+ T cells following *in vitro* activation of aged T cells. (**i**) Quantification of PD-1 expression in CD4+ and CD8+ T cells by flow cytometry (n = 6 biologically independent samples/group). Data shown as mean ± s.e.m.; two-tailed unpaired *t*-test (**b,c,d,f**), two-way ANOVA with a Šidák post hoc test (**i**). Source data

Notably, the known age-related increase in the ratio of myeloid to lymphoid cells^27^ observed in saline-treated aged compared with young control mice was reversed in aged mice that were treated with PF4 (Fig. 3h). Within the myeloid cell population, PF4 treatment restored a more youthful gene signature (Fig. 3i and Extended Data Fig. 4a–c) and decreased the expression of inflammatory signals—such as type I interferon signalling (Fig. 3j), the inflammatory mediators *Lcn2*, *S100a8* and *S100a9* (Fig. 3k and Extended Data Fig. 4g,h), and the anaphylatoxin complement *C3* (Fig. 3k). Further analysis revealed that individual myeloid cell populations (Fig. 3l) were partially returned to young levels in PF4 treated aged mice. Within the lymphoid population, PF4 treatment shifted cells towards a young T cell gene signature (Fig. 3m and Extended Data Fig. 4d–f) and decreased the expression of inflammatory signals and exhaustion markers, such as TNF-mediated signalling (Fig. 3n), the cytotoxic marker *Nkg7* (Fig. 3o) and the transcription factor *Tox* (Fig. 3o). Genes associated with a more naive T cell phenotype that are commonly depressed in aged T cells^28^, including *Sell*, *Dapl1*, *Satb1* and *Foxp1*, were also returned to more youthful levels after systemic PF4 administration relative to the aged saline-treated control mice (Extended Data Fig. 4i–l). Moreover, PF4 treatment returned aged T cell populations to younger levels (Fig. 3p), with a marked decrease in a population of T cells with a gene signature containing high expression of cytotoxic and exhaustion markers that was previously identified as age-associated T effector memory cells^29^ (Fig. 3p and Extended Data Fig. 4m,n). A subset of key cellular and molecular changes in myeloid and lymphoid populations was corroborated by flow cytometry, qPCR and in vitro analysis (Extended Data Fig. 5). These data indicate that PF4 in part restores the cellular composition and molecular signature of the ageing peripheral immune system to a more youthful state.

## PF4 rescues cognition in ageing

**Fig. 4:**
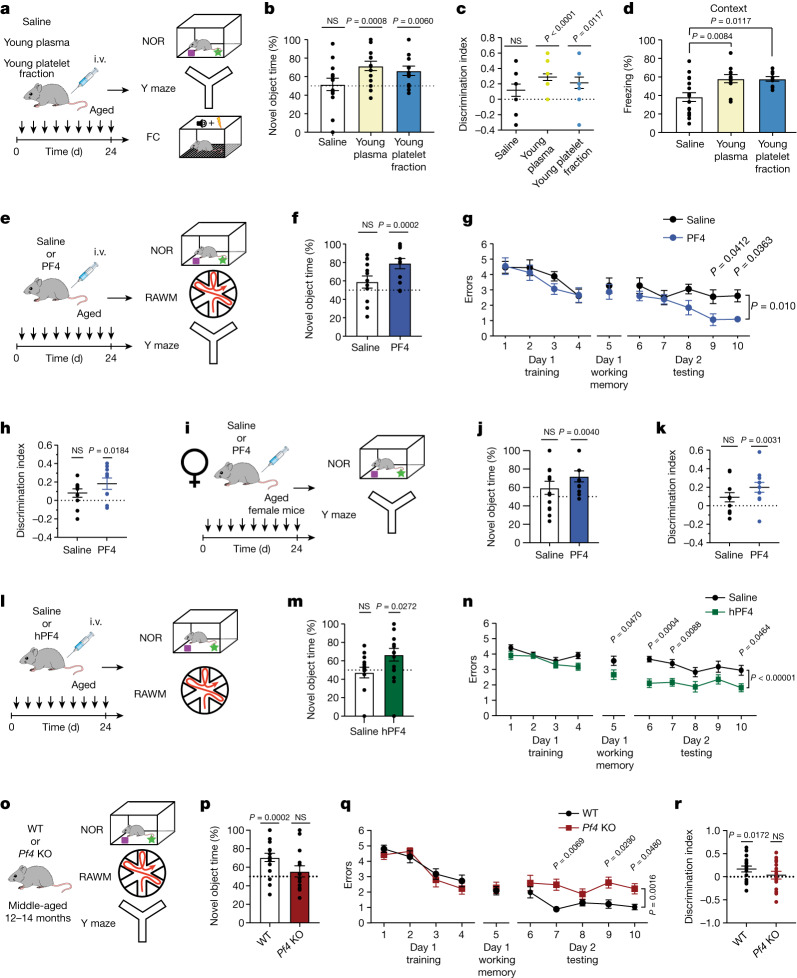
Systemic PF4 improves hippocampal-dependent cognitive function in aged mice. **a**, Schematic of the administration of treatments to aged (20 months) male mice and the cognitive testing timeline. i.v., intravenous; FC, fear conditioning. **b**, Object recognition memory was assessed by NOR as the percentage of time exploring the novel object (*n* = 14 (saline), 15 (young plasma preparation) and 14 (young platelet fraction) mice). **c**, Spatial working memory was assessed using the Y maze as the discrimination index for the novel arm (*n* = 12 (saline), 14 (young plasma preparation) and 14 (young platelet fraction) mice). **d**, Associative fear memory was assessed by contextual fear conditioning as the percentage of time freezing (*n* = 17 (saline), 11 (plasma) and 10 (platelet fraction) mice). **e**,**i**,**l**, Schematics of the administration of saline or mouse PF4 (mPF4) to aged male (**e**) and female (**i**) mice, or hPF4 to aged male mice (**l**) and the cognitive testing timeline. **f**,**j**,**m**, Object recognition memory was assessed by NOR in mPF4-treated aged male (**f**; *n* = 11 (saline) and 14 (PF4) mice) or female (**j**; *n* = 13 (saline) and 11 (PF4) mice) mice, or hPF4-treated aged male mice (**m**; *n* = 15 (saline) and 16 (hPF4) mice). **g**,**n**, Spatial learning and memory was assessed using the RAWM as the number of entry errors in mPF4-treated (**g**; *n* = 12 (saline) and 11 (PF4) mice) and hPF4-treated (**n**; *n* = 19 (saline) and 16 (hPF4) mice) aged male mice. **h**,**k**, Spatial working memory was assessed using the Y maze in mPF4-treated aged male (**h**; *n* = 11 (saline) and 9 (PF4) mice) and female (**k**; *n* = 12 mice per group) mice. **o**, Schematic of cognitive testing of middle-aged (12–14 months) WT and *Pf4*-deficient (*Pf4*-KO) male mice. **p**–**r**, Learning and memory was assessed by NOR (**p**; *n* = 18 WT; 14 *Pf4*-KO mice), RAWM (**q**; *n* = 18 WT; 15 *Pf4*-KO mice) and Y maze (**r**; *n* = 17 WT; 15 *Pf4*-KO mice) testing. Data are mean ± s.e.m. Statistical analysis was performed using two-tailed one-sample *t*-tests (**b**,**c**,**f**,**h**,**j**,**k**,**m**,**p** and **r**), one-way ANOVA with Šidák’s post hoc test (**d**) and two-way ANOVA with Šidák’s post hoc test (**g**, **n** and **q**). Source data

**Extended Data Fig. 6:**
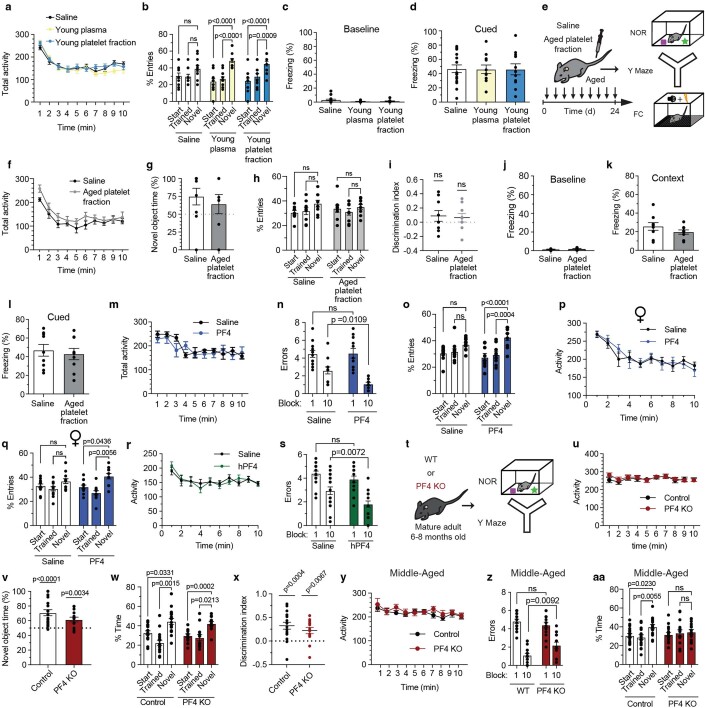
Young platelet factors and PF4 improve hippocampal-dependent cognitive function in aged mice. (**a**) Baseline activity of aged mice (20 months) treated with saline (n = 15), young plasma preparation (n = 15), or the young platelet fraction (n = 16) assessed with open field testing as the number of beam-breaks each minute. (**b**) Spatial working memory assessed by Y Maze as percent of entries in the arms (n = 12 saline; 14 young plasma preparation; 14 young platelet fraction mice). (**c,d**) Baseline freezing behaviour and amygdala-dependent associative fear memory assessed using cued fear conditioning in aged mice treated with saline (n = 17), young plasma preparation (n = 11), or the young platelet fraction (n = 10). (**e**) Schematic illustrates administration of treatments to aged male mice and cognitive testing timeline. (**f**) Baseline activity assessed with open field testing (n = 9 mice/group). (**g**) Object recognition memory assessed by novel object recognition (NOR) as time spent exploring a novel object (n = 9 saline; 7 aged platelet fraction mice). (**h–i**) Spatial working memory assessed by Y Maze as percent of entries (**h**), and the discrimination index for the novel arm (**i**, n = 9 mice/group). (**j–l**) Baseline freezing behaviour and associative fear memory assessed using contextual (**k**) and cued (**l**) fear conditioning as percent time freezing (n = 9 mice/group). (**m,p,r**) Baseline activity assessed with open field testing of mouse PF4 (mPF4) treated aged male (**m**, n = 10 mice/group), or female (**p**, n = 13 saline; 12 PF4 mice) mice, or human PF4 (hPF4) treated aged male mice (**r**, n = 15 saline; 16 hPF4 mice). (**n,s**) Comparison of the number of errors committed during the first and last block of radial arm water maze (RAWM) in mPF4 treated (**n**; n = 12 saline; 11 PF4 mice) and hPF4 treated (**s**; n = 19 saline; 16 hPF4 mice) aged male mice. (**o,q**) Spatial working memory in aged male (**o**, n = 11 saline; 9 PF4 mice) and female mice (**q**, n = 12 mice/group) assessed by Y Maze as percent of entries. (**t**) Schematic illustrates cognitive testing of mature adult (6-8 months old) wild-type (WT) and *Pf4*-deficient (PF4KO) mice. (**u,y**) Baseline activity assessed with open field testing in mature adult (**u**, n = 18 WT; 15 PF4KO mice) and middle-aged male mice (**y**; n = 18 WT; 15 PF4KO mice). (**v**) Object recognition memory assessed by NOR (n = 18 WT; 14 PF4KO mice). (**w-x**) Spatial working memory assessed by Y Maze as percent time in arms (**w**) and the discrimination index for the novel arm (**x**, n = 17 WT; 13 PF4KO mice). (**z**) Comparison of the number of errors committed during the first and last block of RAWM (n = 18 WT; 15 PF4KO mice). (**aa**) Spatial working memory assessed by Y Maze as percent time in arms (n = 17 WT and 15 PF4KO mice). Data shown as mean ± s.e.m.; two-way ANOVA with Šidák’s post-hoc (**a,b,f,h,m,n,o,p,q,r,s,u,w,y,z,aa**), one-way ANOVA with Šidák’s post hoc (**c,d**), two-tailed unpaired *t*-test (**g,j,k,l**), two-tailed one-sample *t*-test (**i,v,x**). Source data

Targeting age-related changes in inflammatory signals in both the aged brain and systemic milieu has been shown to counter, and even reverse, cognitive decline in ageing^23,24,26,30–33^. We reasoned that a decrease in age-related neuroinflammation concurrent with reduced levels of circulating pro-ageing immune factors and rejuvenation of the aged peripheral immune system after systemic administration of platelet factors might improve cognition in aged mice. Hippocampal-dependent learning and memory was assessed using novel object recognition (NOR), forced alternation Y maze and contextual fear conditioning paradigms after intravenous injections with the young blood plasma preparation, the young platelet fraction or saline (Fig. 4a). No differences in overall activity were detected between the treatment groups (Extended Data Fig. 6a,c). During NOR and Y maze testing, aged mice that were treated with either the young blood plasma preparation or the young platelet fraction were biased towards a novel object and the novel arm relative to a familiar condition, whereas saline-treated control mice showed no preference (Fig. 4b,c and Extended Data Fig. 6b). Systemic administration of the young blood plasma preparation or the young platelet fraction increased freezing behaviour during contextual (Fig. 4d), but not cued (Extended Data Fig. 6d), memory testing. As a control, cognitive performance was assessed in aged mice that were administered with either the platelet fraction of aged plasma or saline, and no differences were observed between the treatment groups (Extended Data Fig. 6e–l).

We next examined the potential beneficial effects of systemic PF4 administration on age-related cognitive impairments in hippocampal-dependent learning and memory using NOR, Y maze and radial arm water maze (RAWM) (Fig. 4e). No differences in overall activity were detected between the treatment groups (Extended Data Fig. 6m). During NOR and Y maze testing, aged mice that were treated with PF4 were biased towards a novel object and the novel arm relative to a familiar condition, whereas saline-treated mice showed no preference (Fig. 4f,h and Extended Data Fig. 6o). In the training phase of the RAWM paradigm, all of the mice showed similar spatial learning ability (Fig. 4g). However, aged animals administered with PF4 demonstrated improved learning and memory for the platform location during the testing phase of the task compared with the aged saline-treated control mice (Fig. 4g and Extended Data Fig. 6n). Having conducted our behavioural analysis up to this point in male mice, we examined the effect of systemic PF4 administration on cognitive function in aged female mice using NOR and Y maze testing and observed cognitive improvements across sexes (Fig. 4i–k and Extended Data Fig. 6p,q).

To begin to investigate the translational potential of PF4, aged male mice were systemically administered with human PF4 (hPF4) derived from human platelets (5 µg ml^−1^), and hippocampal-dependent learning and memory was assessed using NOR and RAWM testing (Fig. 4l). No adverse effects or differences in overall activity were observed between the hPF4 and saline treatment groups (Extended Data Fig. 6r). Aged mice treated with hPF4 were biased towards a novel object relative to a familiar object, whereas saline-treated mice showed no preference (Fig. 4m). All of the mice showed similar spatial learning ability during RAWM training (Fig. 4n). However, aged animals administered with hPF4 demonstrated improved learning and memory for the platform location during the testing phase of the task compared with the aged saline-treated control mice (Fig. 4n and Extended Data Fig. 6s).

Finally, we investigated whether the loss of PF4 negatively impacted cognition before the typical onset of age-related cognitive decline. Hippocampal-dependent learning and memory was assessed in mature adult and middle-aged *Pf4-*KO mice using NOR, Y maze and RAWM testing (Fig. 4o and Extended Data Fig. 6t). No differences in overall activity were detected between *Pf4-*KO and littermate WT controls at any age (Extended Data Fig. 6u,y). No cognitive deficits were observed in mature adult *Pf4-*KO and control mice (Extended Data Fig. 6v–x). However, whereas middle-aged control mice remained biased toward a novel object and the novel arm relative to a familiar condition during NOR and Y maze testing, middle-aged *Pf4-*KO mice no longer exhibited a preference (Fig. 4p,r and Extended Data Fig. 6aa). All of the mice showed similar spatial learning ability during RAWM training (Fig. 4q). However, middle-aged *Pf4-*KO mice demonstrated impaired learning and memory for the platform location during the testing phase of the task (Fig. 4q and Extended Data Fig. 6z). These behavioural data indicate that systemic PF4 administration enhances cognitive function in aged mice, whereas the loss of PF4 accelerates cognitive decline in an age-dependent manner by middle-age.

## CXCR3 mediates, in part, the benefits of PF4

**Fig. 5:**
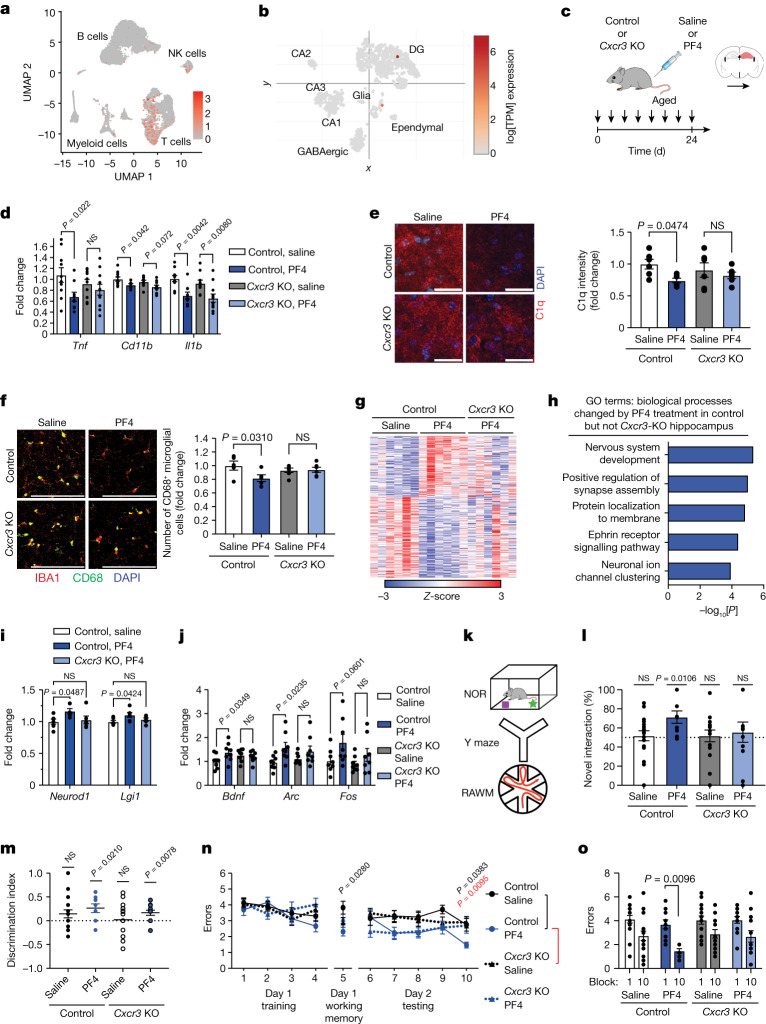
CXCR3 mediates, in part, the benefits of systemic PF4 on the aged hippocampus. **a**,**b**, *Cxcr3* expression in spleen (**a**) and hippocampus (**b**) clusters was analysed using single-cell and single-nucleus RNA-seq^40^, respectively. DG, dentate gyrus; TPM, transcripts per million. **c**, The timeline of saline or PF4 administration to aged (19–21 months) *Cxcr3*-deficient (*Cxcr3*-KO) and littermate control (WT/heterozygous) mice. **d**, qPCR analysis of neuroinflammation-related gene expression relative to *Gapdh* in the aged hippocampus (*n* = 10 (control, saline), 9 (control, PF4), 10 (*Cxcr3*-KO, saline) and 11 (*Cxcr3*-KO, PF4) mice). **e**,**f**, Representative images and quantification of C1q signal intensity (**e**; *n* = 6 mice per group) and IBA1^+^ and CD68^+^ cells (**f**; *n* = 5 (control, saline), 5 (control, PF4), 6 (*Cxcr3*-KO, saline) and 6 (*Cxcr3*-KO, PF4) mice) in the dentate gyrus of the aged hippocampus. **g**–**i**, RNA-seq analysis of aged hippocampi from saline- and PF4-treated control and PF4-treated *Cxcr3*-KO mice (*n* = 6 mice per group). Scale bars, 25 μm (**e**) and 100 μm (**f**). **g**, Significant DEGs (*P* < 0.01) from PF4-treated mice relative to saline-treated control mice. **h**, GO terms associated with DEGs after PF4 treatment in control mice, but not *Cxcr3*-KO mice. **i**, The fold change (FPKM) in synaptic-plasticity-related genes. **j**, qPCR analysis of synaptic-plasticity-related gene expression relative to *Gapdh* in the aged hippocampus (*n* = 8 mice per group). **k**, Schematic of cognitive testing. **l**, Object recognition memory was assessed using NOR as the percentage time spent exploring the novel object (*n* = 17 (control, saline); 9 (control, PF4), 15 (*Cxcr3*-KO, saline) and 11 (*Cxcr3*-KO, PF4) mice). **m**, Spatial working memory was assessed using the Y maze as the discrimination index for the novel arm (*n* = 17 (control, saline), 9 (control, PF4), 16 (*Cxcr3*-KO, saline) and 13 (*Cxcr3*-KO, PF4) mice). **n**,**o**, Spatial learning and memory was assessed using the RAWM as the number of entry errors (*n* = 17 (control, saline), 10 (control, PF4); 17 (*Cxcr3*-KO, saline) and 13 (*Cxcr3*-KO, PF4) mice). Data are mean ± s.e.m. Statistical analysis was performed using two-tailed one-sample *t*-tests (**l** and **m**), one-way ANOVA with Šidák’s post hoc test (**d**–**f**, **i** and **j**), Fisher’s exact tests (**h**), two-way ANOVA with Tukey’s post hoc test (**n**) and three-way ANOVA with Šidák’s post hoc test (**o**). Source data

**Extended Data Fig. 7:**
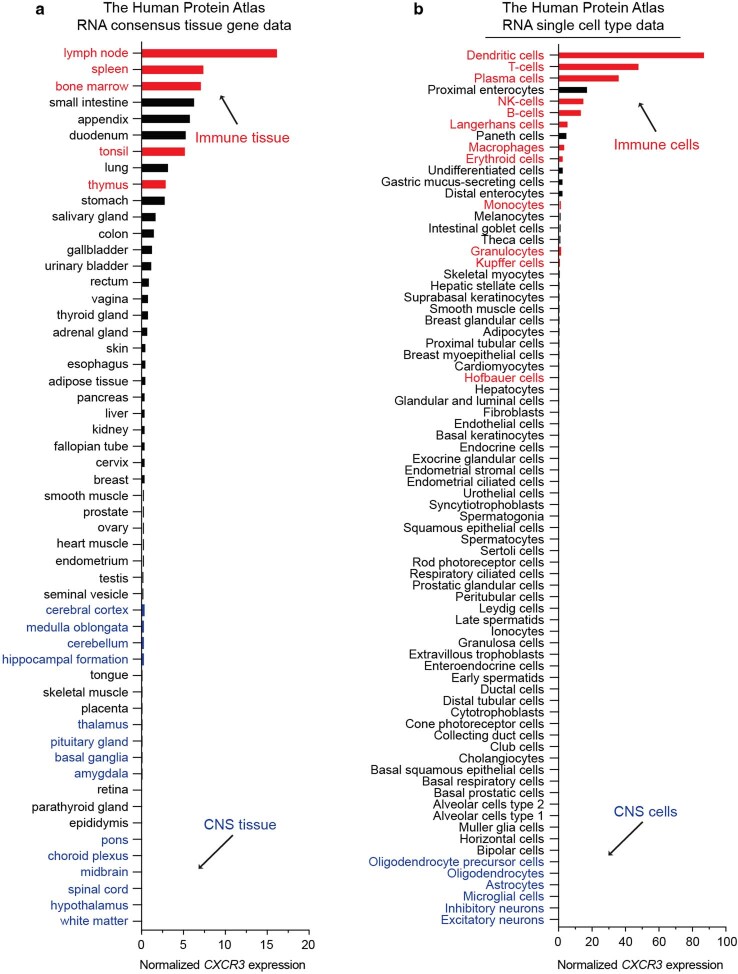
Expression of *CXCR3* across human tissues and cells. (**a-b**) *CXCR3* expression levels in human tissues and cells from previously published sequencing data (The Human Protein Atlas). (**a**) Consensus transcript expression levels in 54 tissues based on transcriptomics data from HPA and GTEx. The consensus normalized expression value is calculated as the maximum value for each gene in the two data sources. (**b**) Transcript expression levels of *CXCR3* in 76 cell types from 26 datasets. Red bars indicate immune tissues and cells, and blue bars indicate CNS regions and cells. Underlying data are available at v21.proteinatlas.org and downloadable at http://www.proteinatlas.org/ENSG00000186810.tsv. Source data

PF4 acts through multiple receptors and molecular partners to induce various downstream signalling cascades^34^. Of these, the chemokine receptor CXCR3 has been ascribed a predominant role in mediating the actions of PF4 in humans, with emerging evidence also indicating a functional role in mice^35–39^. Thus, to gain mechanistic insights, we investigated CXCR3 as a possible intermediary of the beneficial effects of systemic PF4 on the aged hippocampus. First, we assessed expression of CXCR3 using our CITE-seq data (Fig. 5a) and publicly available mouse^40^ and human RNA-seq datasets (The Human Protein Atlas) (Fig. 5b and Extended Data Fig. 7a,b), and detected prominent expression in lymphoid and myeloid immune cell populations with nearly no detection in the brain. Given that the PF4–HiBiT data suggest a peripheral mechanism of action (Fig. 3b and Extended Data Fig. 2a), we examined whether the loss of CXCR3 affected the benefits of systemic PF4.

**Extended Data Fig. 8:**
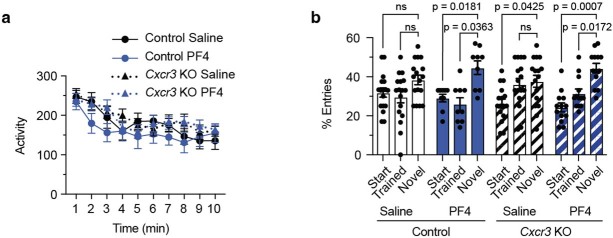
Systemic administration of PF4 in aged Cxcr3 KO and control mice does not alter activity. (**a**) Baseline activity of aged control and *Cxcr3* KO mice treated with saline or PF4 assessed with open field testing as the number of beam-breaks each minute (n = 12 control/saline; 11 control/PF4; 17 CXCR3KO/saline; 16 CXCR3KO/PF4 mice). (**b**) Spatial working memory assessed by Y Maze as percent of entries in the start, trained, and novel arms (n = 17 control/saline; 9 control/PF4; 16 CXCR3KO/saline; 13 CXCR3KO/PF4 mice). Data shown as mean±s.e.m.; two-way analysis of variance (ANOVA) with Šidák’s correction for multiple comparisons (**a,b**). Source data

Aged *Cxcr3*-KO or littermate control (WT/heterozygous) mice were systemically administered with PF4 or saline (Fig. 5c), and hippocampal neuroinflammation, transcriptional changes and cognitive function were assessed. We detected decreased expression of the pro-inflammatory genes *Tnf*, *CD11b* and *Il1b* (Fig. 5d), reduced C1q levels (Fig. 5e) and a decrease in activated CD68^+^ microglia (Fig. 5f) in the hippocampus of aged control mice administered with PF4 compared with saline. However, the effects of systemic PF4 on neuroinflammation markers were in part blunted in aged *Cxcr3*-KO mice (Fig. 5d–f). We next performed RNA-seq analysis of the hippocampus of aged control and *Cxcr3*-KO mice after systemic PF4 administration. GO analysis of genes that were differentially expressed in control mice after PF4 administration, but not altered in PF4-treated *Cxcr3*-KO mice, indicate mitigation of molecular changes associated with nervous system development and synaptic processes (Fig. 5g,h and Supplementary Table 4). Loss of CXCR3 abrogated the expression of synaptic-plasticity-related markers, such as *Lgi1* and *Neurod1* (Fig. 5i). We also detected abrogation of CREB-mediated synaptic plasticity genes, including *Bdnf, Arc* and *Fos* on the basis of qPCR analysis (Fig. 5j). Last, we assessed hippocampal-dependent cognitive function using the NOR, Y maze and RAWM paradigms (Fig. 5k). No differences in overall activity were detected regardless of the treatment or genotype (Extended Data Fig. 8a). During NOR and Y maze testing, PF4-treated control mice were biased towards a novel object and the novel arm relative to a familiar condition (Fig. 5l,m and Extended Data Fig. 8b). Whereas preference for the novel arm persisted in PF4-treated *Cxcr3*-KO mice (Fig. 5m and Extended Data Fig. 8b), no preference was observed for a novel object (Fig. 5l). All of the mice showed similar spatial learning ability in the training phase of the RAWM test (Fig. 5n). Control mice that were administered with PF4 demonstrated improved spatial learning and memory for the platform location during the testing phase of the task compared with the saline-treated control mice (Fig. 5n,o). However, cognitive enhancements in spatial learning and memory were blunted by the end of the testing phase in PF4-treated *Cxcr3*-KO mice (Fig. 5n,o). These data indicate that the loss of CXCR3 mitigates, in part, the benefits of systemic PF4 administration on the aged hippocampus, and posit additive mechanisms of action that combine to contribute to the full benefit of systemic PF4 on the aged brain.

## Discussion

Cumulatively, our data demonstrate that platelet factors transfer the restorative effects of young blood on immune and cognitive function to the aged brain. We identified the platelet-derived chemokine PF4 as a pro-youthful factor that attenuates age-related neuroinflammation, elicits synaptic-plasticity-related molecular changes and rescues hippocampal-dependent learning and memory in aged mice. We further implicate decreased levels of circulating pro-ageing immune factors and rejuvenation of the ageing peripheral immune system in the restorative effects of PF4 on the aged brain. Mechanistically, we demonstrate that CXCR3, in part, mediates the cellular, molecular and cognitive benefits of systemic PF4 on the aged brain. Ultimately, our data identify circulating platelet factors as potential therapeutic targets to abate inflammation and rescue cognition in old age.

The beneficial effects of PF4 on the aged brain are probably the result of changes in multiple downstream cellular and molecular signalling cascades, as posited by our in vivo CXCR3 data, and necessitate further investigation. Notwithstanding, PF4 has been shown to increase in the blood after multiple systemic rejuvenating interventions, such as neutral blood exchange in aged mice^16^, suggesting a potential common mechanism of action across broad rejuvenating strategies. Although PF4 must ultimately induce neuronal changes to improve cognitive functions, our findings point to intermediary roles of the peripheral immune system in young-blood-mediated restoration of the aged hippocampus. Indeed, a prominent role is emerging for the peripheral immune system as a regulator of brain ageing. Age-related cellular changes in myeloid cells^31,41^, T cells^42,43^ and natural killer cells^44^ are implicated as drivers of decreased regenerative capacity, increased senescence and cognitive impairments in the ageing brain. Moreover, we and others have demonstrated that the aged haematopoietic system promotes hippocampal-dependent cognitive impairments^24,32^, in part through increased systemic levels of pro-ageing immune factors such as CCL2 and CyPA^25,26^. By contrast, reconstituting a young haematopoietic system^33^, restoring a more youthful metabolism in aged peripheral myeloid cells^31^, and targeting systemic pro-ageing immune factors such as CyPA in blood^25,26^ ameliorates cognitive impairments in aged mice. In this context, our results suggest that maladaptive inflammation and cognitive decline in the aged brain can potentially be reversed by therapeutically leveraging platelet factors to rejuvenate the cellular composition and molecular signature of the ageing peripheral immune system. Given the strong association between increased inflammation and age-related neurodegenerative diseases^11,45–47^, such as Alzheimer’s disease, our data further raise the possibility that the beneficial effects of platelet factors may extend more broadly to dementia-related disorders in older people.

## Methods

### Animal models

The C57BL/6 mouse line was used for all of the experiments (The Jackson Laboratory and National Institutes of Aging). Homozygous *Pf4*-KO mice were previously generated and characterized as described^48^. *Pf4*-KO mice were a gift from M. Anna Kowalska. Heterozygous mice were bred to generate *Pf4*-KO and WT littermate controls. *Cxcr3-*deficient mice (*Cxcr3*-KO) were created and characterized by Deltagen. Homozygous *Cxcr3-*KO mice were acquired from The Jackson Laboratory (005796). Breeding mates consisting of heterozygous females and hemizygous males were established to produce experimental male *Cxcr3*-KO and WT controls, and female *Cxcr3-*KO and heterozygous controls. All other studies were performed with male mice, unless indicated otherwise. The numbers of mice used to result in statistically significant differences was calculated using standard power calculations with *α* = 0.05 and a power of 0.8. We used an online tool (http://www.stat.uiowa.edu/~rlenth/Power/index.html) to calculate power and sample size on the basis of experience with the respective tests, variability of the assays and interindividual differences within groups. Animals used for each individual experiment were from the same vendor and aged together. All animals from Jackson Laboratories were acquired at 2 months of age and were aged in-house. The mice were moved to a new location for behavioural assessment. The two locations where we conducted behavioural analysis are on the UCSF Parnassus campus: the UCSF Rehabilitation Behaviour Core and the Villeda Lab Behavioural Suite. Mice were housed under specific-pathogen-free conditions under a 12 h–12 h light–dark cycle, with humidity maintained at 30–70% and temperature at 68–79 °F (20–26 °C). All animal handling and use was performed in accordance with institutional guidelines approved by the University of California San Francisco IACUC.

### Animal plasma collection, platelet fraction preparation and systemic administration

Mouse blood was collected by intracardial bleed at time of euthanasia from young (3 months old) and aged (20 months old) mice. Blood was collected with EDTA, followed by centrifugation at 1,000*g* for plasma preparation. For western blot and ELISA analysis, plasma was aliquoted and stored at −80 °C until use. Before systemic administration, plasma was dialysed using 3.5 kDa D-tube dialyzers (EMD Millipore) in PBS to remove EDTA. For platelet fraction preparation, plasma was centrifuged at 20,000*g*, the supernatant was discarded and the pelleted platelet component was resuspended in an equivalent volume of saline. Aged mice were systemically treated with saline, plasma or the platelet fraction (100 μl per injection) through intravenous tail vein injection 8 times over 24 days. Likewise, saline or PF4 (5 μg ml^−1^) was systemically administered to mice (100 μl per injection) through intravenous tail vein injection 8 times over 24 days. Recombinant mouse PF4 (CHM-245, ProSpec) and human-platelet-derived PF4 (CHM-234, ProSpec) were dissolved in sterile ultrapure water at 100 µg ml^−1^ (according to the manufacturer’s instructions), before final dilution in saline.

### Tissue collection

Mice were anesthetized with 87.5 mg per kg ketamine and 12.5 mg per kg xylazine and transcardially perfused with ice-cold phosphate-buffered saline. Tissues were removed and processed for subsequent analysis. To process the brains, either the hippocampus was subdissected and snap-frozen or the whole brain was fixed in phosphate-buffered 4% paraformaldehyde, pH 7.4 at 4 °C for 48 h before cryoprotection with 30% sucrose.

### RNA isolation and bulk RNA-seq

RNA was isolated from either the whole hippocampus or microglia isolated from the hippocampus. Microglia were isolated from hippocampi by enzymatic and mechanical dissociation using the Neural Tissue Dissociation Kit (P) (Miltenyi Biotec) according to the manufacturer’s instructions. Myelin was depleted using Myelin Removal Beads (Miltenyi Biotec). Subsequently, microglia were captured using CD11b microbeads (Miltenyi Biotec). Total RNA was isolated from samples by lysis using TRIzol Reagent (Thermo Fisher Scientific), separation with chloroform and precipitation with isopropyl alcohol, according to the manufacturer’s instructions. After RNA isolation, RNA-seq libraries were constructed using the Smart-Seq2 protocol^49^ with modifications. In brief, 1 ng high-quality RNA was reverse-transcribed using SuperScript II (Life Technologies, 18064-014) with a poly-dT anchored oligonucleotide primer, and a template switching oligonucleotide primer that generated homotypic PCR primer binding sites. The cDNA underwent 10 rounds of PCR amplification using KAPA HiFi Hotstart (Kapa Biosystems, KK2601), followed by Ampure bead (Agencourt) clean-up. The quality of the amplified cDNA was tested by qPCR analysis of *Gapdh* and nucleic acid quantification. A total of 1 ng of high-quality amplified cDNA was fragmented with the Tn5 transposase from the Illumina Nextera kit (FC-131-1096) to a median size of around 500 bp. The fragmented library was amplified with indexed Nextera adapters (FC-131-1002) using 12 rounds of PCR. The final libraries were purified with Ampure beads and quantified using the qPCR Library Quantification Kit (Kapa Biosystems, KK4824). Libraries were pooled for sequencing on the Illumina HiSEq 2500 system (paired-end reads 2 × 100 bp). Alignment of RNA-seq reads to the mouse mm10 transcriptome was performed using STAR (v.2.7.3a)^50^ using the ENCODE standard options, read counts were generated using RSEM (v.1.3.1) and differential expression analysis was performed in R (v.3.6.1) using the DESeq2 package (v.1.38.0)^51^ (detailed pipeline v.2.0.1 and options are available at GitHub (https://github.com/emc2cube/Bioinformatics/)). Genes significantly changed after treatment with both plasma and the platelet fraction were determined using a nominal *P* < 0.05, and significance in microglia after systemic PF4 administration was determined with a nominal *P* < 0.01. GO term enrichment analysis was performed using Enrichr^52^ (GO Biological Process 2018). Heat maps were generated using Morpheus (https://software.broadinstitute.org/morpheus/).

### RT–qPCR

To quantify mRNA expression levels, equal amounts of cDNA were synthesized using the High-Capacity cDNA Reverse Transcription kit (Thermo Fisher Scientific, 4368813), then mixed with SYBR Fast mix (Kapa Biosystems) and primers. *Gapdh* was amplified as an internal control. RT–qPCR was performed in the CFX384 Real Time System (Bio-Rad). Each sample and primer set were run in triplicates and relative expression levels were calculated using the ${2}^{-\Delta \Delta {C}_{{\rm{t}}}}$ method^53^.

### Immunohistochemistry

Tissue processing and immunohistochemistry was performed on free-floating sections according to standard published techniques^26^. Cryoprotected brains were sectioned coronally at 40 μm with a cryomicrotome (Leica Camera). Free-floating coronal sections (40 μm) were incubated overnight with anti-IBA1 (1:1,000, Wako, 0191741; or 1:1,000, Synaptic Systems, 234-004), rat anti-CD68 (FA-11, 1:250, Bio-Rad MCA1957), rabbit anti-C1q (1:500, Abcam ab182451) and anti-phosphorylated-CREB (Ser133) (1:2,500, Millipore 06-519) primary antibodies. Labelling was revealed using secondary antibodies (donkey anti-rabbit conjugated Alexa Fluor 555 (1:750, Life Technologies, A31572), donkey anti-rat conjugated Alexa Fluor 647PLUS (1:750, Invitrogen, A48272) donkey anti-guinea pig conjugated Alexa Fluor 488 (1:750, Jackson ImmunoResearch, 706-545-148) and goat anti-rabbit, biotinylated (1:500, Vector, BA-1000)). Labelling of biotinylated antibodies was revealed using the Vectastain Elite ABC-HRP Detection Kit (Vector, PK-6100) with diaminobenzidine (Sigma-Aldrich, D5905). Sections were imaged using confocal microscopy (Zeiss LSM800 or Zeiss LSM900) or bright-field microscopy (Keyance). Individual cell numbers and intensity in the dentate gyrus was quantified across 3–4 sections per animal using ImageJ.

### Collection and preparation of human platelet-rich plasma

Blood was collected from healthy young men (aged 20–35 years) or healthy older men (aged 60–75 years), who volunteered for either a cross-sectional or non-randomized single-arm study at the UCSF Human Performance Center. Samples used in this experiment were from the cross-sectional baseline timepoint only. The participants were asked to complete detailed surveys about their health habits, including physical activity, and donate a fasting blood sample. This study was approved by the Institutional Review Board of UCSF. Samples were drawn from consenting participants by the UCSF Clinical and Translational Science Institute Blood laboratory, and immediately transported to the Villeda laboratory for processing. One 6 ml aliquot of blood was centrifuged at 500*g* for 8 min (4 °C) and the plasma was collected. The plasma was aliquoted and centrifuged again at 700*g* for 17 min (12 °C). Platelet-poor plasma was removed as the top 70% of the solution and the remaining solution was used to resuspend the pellet as platelet-rich plasma. The samples were aliquoted and stored at −80 °C until use.

### Western blot analysis and ELISA

For Western blot analysis, samples were combined with RIPA lysis buffer (Abcam, ab156034) with complete protease inhibitor (4693116001, Sigma-Aldrich) and phosphatase inhibitor (Thermo Fisher Scientific, 78420). Subsequently, the samples were mixed with 4× NuPage LDS loading buffer (Invitrogen, NP0008), loaded onto an SDS polyacrylamide gel (Invitrogen) and transferred onto a nitrocellulose membrane. Equal loading of samples was confirmed using Ponceau S solution (Sigma-Aldrich, P7170) and membranes were imaged with the ChemiDoc System (Bio-Rad). The blots were blocked in 5% milk in Tris-buffered saline with Tween-20 and incubated with anti-GAPDH (6C5, 1:5,000, Abcam, ab8245) goat anti-mPF4 (1 µg ml^−1^, R&D Systems, AF595), mouse anti-hPF4 (170138, 0.5 µg ml^−1^, R&D Systems, MAB7952), mouse anti-thrombospondin-1 (A6.1, 1:200, Santa Cruz, sc-59887, C2519) or rabbit anti-cyclophilin A (1:200, ENZO Life Sciences, BML-SA296-0100). Horseradish-peroxidase-conjugated secondary antibodies (donkey anti-goat conjugated HRP (1:2,000, Invitrogen, A15999), goat anti-mouse conjugated HRP (1:2,000, Millipore, AP124P), and donkey anti-rabbit conjugated HRP (1:2,000, GE Healthcare, NA934V)) and an ECL kit (GE Healthcare) were used to detect protein signals. Developed membranes were imaged using the ChemiDoc System (Bio-Rad). Selected images were exported and quantified using ImageJ (v.2.0.0). Plasma protein levels were measured by ELISA for PF4 (Mouse CXCL4/PF4 Quantikine ELISA Kit; R&D Systems; MCX400), CCL2 (Mouse CCL2/JE/MCP-1 Quantikine ELISA Kit; R&D Systems; MJE00B), TNF (mouse TNF Quantikine ELISA Kit; R&D Systems; MTA00B) and β_2_-microglobulin (Cloud-Clone Corp; SEA260Mu) according to the manufacturer’s protocol.

### HDTVI of HiBiT plasmids

To detect the localization of HiBiT-tagged PF4 and TRF in various mouse tissues, mice were hydrodynamically injected with GFP, PF4-HiBiT or TRF-HiBiT constructs as previously described^7,54^. To generate plasmids, RNA was isolated from mouse peripheral blood mononuclear cells (PBMCs) or liver using TRIzol reagent (Thermo Fisher Scientific, 15596026) and the PureLink RNA Mini Kit. RNA was reverse-transcribed using the High-Capacity cDNA Reverse Transcription Kit (Thermo Fisher Scientific, 4368813) and oligo dT primers (Promega, C1101). The following primers were used for PCR amplification of the *Pf4* coding sequences and partial 3′ and 5′ untranslated regions from the PBMC cDNA library: forward, CACCAGTGGCACCCTCTTGACAT; and reverse, GGCAGCTGATACCTAACTCTCC. *Trf* was amplified from liver cDNA using the following primers: forward, CACCAGCGGGTCGGTCTGTACTC; and reverse, CAGTGGCAACCCACCTCTTG. *Pf4* and *Trf* ORFs were cloned into the pENTR D-TOPO vector (Thermo Fisher Scientific, K240020) and sequence verified using M13F and M13R sequencing primers. Restriction enzyme sites (Nhel and EcoR1 for *Pf4* and Nhel and Mfel for *Trf*), a Kozak sequence and a C-terminal HiBiT tag were added during an additional PCR amplification step. The resulting PCR fragments were cloned into a mammalian expression plasmid using the designated restriction sites. The bicistronic plasmid vectors expressed *Pf4* or *Trf* and an IRES eGFP reporter using a CMV promoter. An empty IRES eGFP construct based on the same plasmid was used as a control. All coding plasmid sequences were verified by Sanger sequencing. Endotoxin-free plasmid kits were used for plasmid preparation before in vivo use. To perform HDTVI of constructs, plasmid DNA (50 μg) was suspended in 3 ml saline and injected in the tail vein in 5–7 s in mice. At 24 h after HDTVI, the mice were euthanized and plasma was collected by intracardial bleed. After perfusion, the hippocampus, cortex, cerebellum and liver were dissected, snap-frozen and lysed in RIPA lysis buffer (Abcam, ab156034) with cOmplete protease inhibitor (Sigma-Aldrich, 4693116001) and phosphatase inhibitor (Thermo Fisher Scientific, 78420). A total of 20 μg of protein from each sample was loaded in duplicate in an opaque 96-well plate (Corning, 353296). HiBiT luminescence was measured on the Cytation 5 (BioTek) using the Nano-Glo HiBiT Lytic Detection System (Promega, N3030) according to the manufacturer’s instructions.

### Splenocyte isolation

Splenocytes were isolated from young saline-treated, aged saline-treated and aged PF4-treated mice. For collection of splenocytes, spleens were removed, mechanically dissociated with a syringe plunger over a 70 μm cell strainer and washed with 10 ml of ice-old RPMI medium with 2% FBS. Cells were centrifuged and RBC lysis was performed (155 mM NH_4_Cl, 1 mM KHCO_3_ and 0.1 mM EDTA). Subsequently, cells were washed and resuspended in staining buffer.

### CITE-seq

CITE-seq analysis was performed on splenocytes isolated from five mice per group. Cells from all five mice from each group were pooled for CITE-seq antibody labelling, as previously described^55^ (detailed methods are available online (https://citeseq.files.wordpress.com/2019/02/cite-seq_190213.pdf)). In brief, the samples were blocked with TruStain fcX (BioLegend) for 10 min on ice. After the blocking step, the samples were incubated on ice with Total-seqB antibodies purchased from BioLegend (Extended Data Table 1). After 30 min of labelling, samples were washed three times and filtered through a 40 µm cell strainer before delivering the prepared samples to the UCSF-IHG Genomics Core for analysis with the 10x Genomics Chromium Single Cell Expression Solution 3′ kit with Feature Barcode Technology (v.3.1). The Genomics Core prepared cells for 10x Genomics Chromium single-cell capture. 10,000 cells were loaded per sample. cDNA libraries were prepared according to the standard 10x Genomics protocols. The final library pool was sequenced to a depth of 30,000 cDNA reads per cell and 3,000 ADT reads per cell on the NovaSeq 6000 S2 system. The raw base sequence calls were demultiplexed into sample-specific cDNA and ADT files with bcl2fastq/mkfastq sample sheet using Cell Ranger (10x Genomics). CITE-seq analysis and statistical analysis of raw FASTQ files were processed using the Cell Ranger software package (10x Genomics) for the RNA expression matrix and CITE antibody counts matrix. The data were combined using the Cell Ranger aggrpipeline (10x Genomics). Downstream single-cell analysis was performed using the R package Seurat^56^. Data were processed to remove doublets and unwanted sources of variation by removing cells with more than 5,000 and fewer than 300 genes per cell and regressing on number of UMIs. Genes expressed in fewer than three cells were filtered out. Cells with a percentage of mitochondrial genes of higher than 10% were removed. The matrices of data were log-normalized in a sparse data matrix and PCA was applied to reduce dimensionality. The first 20 PCA components were used to cluster cells by Louvain clustering implemented in Seurat while UMAP plots were independently generated to aid in 2D representation of multidimensional data independent of the clustering. Log-normalized gene expression data were used for visualizations with violin plots, UMAP plots and generation of heat maps.

### Flow cytometry

Flow cytometry analysis of platelets was performed on whole blood and the platelet fraction of the plasma preparation. After collection, blood was diluted 1:10 in 250 mM EDTA. Platelets were labelled with anti-CD61 PE (2C9.G2, HMβ3-1, 1:50, BioLegend, 104308) at 4 °C. Cells were washed and resuspended in PBS for analysis with the BD LSR II Flow Cytometer. Flow cytometry analysis of splenocytes was performed, as previously described^57^. Cells were labelled with two independent panels to assess specific populations of myeloid cells and lymphocytes. Antibodies for the myeloid panel were as follows: CD45-BUV395 (30-F11, 1:200, BD, 564279), CD3-APC (17A2, 1:200, Tonbo Biosciences, 20-0032-U025), B220-APC (RA3-6B2, 1:200, BioLegend, 103211), CD49b-APC (DX5, 1:200, eBioscience, 50-112-9698), Ly6G-BV711 (1A8, 1:200, BioLegend, 127643), I-A/I-E Alexa Fluor 700 (M5/114.15.2, 1:200, BioLegend, 107621), F4/80-PeCy7 (BM8, 1:200, eBioscience, 25480182) and CD11b-BV650 (M1/70, 1:200, BioLegend, 101239). Antibodies for the lymphoid panel were as follows: CD45-BV711 (30-F11, 1:200, BD, 563709), B220-PeCy5 (RA3-6B2, 1:200, eBioscience, 15-0452-82), CD4-PeCy7 (RM4-5, 1:200, eBioscience, 25-0042-82), CD3-APC (17A2, 1:200, Tonbo Biosciences, 20-0032-U025), CD8a-Pacific Blue (5H10, 1:200, Thermo Fisher Scientific, MCD0828), CD62L-PerCP-Cyanine5.5 (MEL-14, 1:100, Tonbo Bioscience, 65-0621-U100) and CD44-APC eFluor 780 (IM7, 1:100, eBioscience, 47-0441-82). In brief, 1 × 10^6^ cells were blocked with FBS and stained at 4 °C. Thereafter, the cells were washed, fixed with 4% paraformaldehyde solution, washed and resuspended in PBS for storage until analysis using the BD LSR II Flow Cytometer.

### T cell culture and activation

For in vitro experiments, untouched T cells were isolated from aged mouse spleens using the Pan T Cell Isolation Kit II, mouse (Miltenyi Biotec), according to manufacturer’s instructions. Isolated splenocytes were resuspended in staining buffer (PBS, pH 7.2, 0.5% BSA and 2 mM EDTA) before addition of biotin-conjugated antibody. After a 5 min incubation at 4 °C, additional buffer and anti-biotin microbeads were added to the solution. Cells were incubated for 10 min and the suspension was applied to a washed LS column on a QuadroMACS Separator (Miltenyi Biotec), while the unlabelled flow-through was collected as the pan T cell fraction. Cells were washed and plated in culture medium (RPMI with 10% FBS and 55 μM BME). Subsequently, T cells were stimulated (Dynabeads Mouse T-Activator CD3/CD28 for T-Cell Expansion and Activation; Thermo Fisher Scientific) with or without recombinant mouse PF4 (1 μg ml^−1^; Prospec). Cells were incubated for 3 days before collection for analysis. Flow cytometry analysis of the cells was performed using the following antibodies: CD3-eFluor 660 (17A2, 1:100, eBioscience, 50-0032-82), CD4-PE-Cyanine7 (RM4-5, 1:200, eBioscience, 25-0042-82), CD8a-PE (53-6.7, 1:100, BioLegend, 100708) and CD279/PD-1-FITC (29F.1A12, 1:200, BioLegend, 135214). In brief, 1 × 10^6^ cells were blocked with FBS and stained at 4 °C. Thereafter, the cells were fixed with 4% paraformaldehyde solution, washed and resuspended in PBS for storage until analysis with the flow cytometer (BD Accuri C6 Plus).

### NOR

The NOR task was performed as previously described^7^. On day one (the habituation phase), mice performed open field testing by exploring an empty arena for 10 min. Infrared photobeam breaks were recorded and movement metrics were analysed using the MotorMonitor software (Kinder Scientific). On day two (the training phase), two identical objects were placed into the habituated arena, and the mice were allowed to explore for 5 min. On day three (the testing phase), one object was replaced with a novel object, and the mice were allowed to explore for 5 min. The time spent exploring each object was quantified using the Smart Video Tracking Software (Panlab; Harvard Apparatus). Two different sets of objects were used. To control for any inherent object preference, half of the mice were exposed to object A as their novel object and half to object B. To control for any potential object-independent location preference, the location of the novel object relative to the trained object was also counterbalanced. To determine the percentage of time with the novel object, we calculate (time with novel object)/(time with trained object + time with novel object) × 100. Mice that did not explore both objects during the training phase were excluded from the analysis.

### Y maze

The Y Maze task was conducted as previously described^7^. During the training phase, the mice were placed into the start arm facing the wall and were allowed to explore the start and trained arm for 5 min, while the entry to the 3rd arm (novel arm) was blocked. The maze was cleaned between each mouse to remove odour cues, and the trained arm was alternated between mice. After training, the mouse was returned to its home cage. After 45 min, the mouse was returned to the start arm and was allowed to explore all three arms for 5 min. The number of entries and the time spent in each arm was quantified using the Smart Video Tracking Software (Panlab; Harvard Apparatus). The percentage of entries in each arm was defined as the number of entries in each arm divided by the total number of entries in all arms during the first minute of the task. The discrimination index was quantified by (novel arm − trained arm)/(novel arm + trained arm). Mice that did not perform three entries during the first minute of testing were excluded.

### Fear conditioning

In this task, mice learned to associate the environmental context (fear conditioning chamber) with an aversive stimulus (mild foot shock; unconditioned stimulus) enabling testing for hippocampal-dependent contextual fear conditioning. To assess amygdala-dependent cued fear conditioning, the mild foot shock was paired with a light and tone cue (conditioned stimulus). Freezing behaviour was used as a readout of conditioned fear. Specific training parameters were as follows: tone duration of 30 s; level of 70 dB, 2 kHz; shock duration of 2 s; intensity of 0.6 mA. This intensity is not painful and can easily be tolerated but will generate an unpleasant feeling. On the training day (day 1), each mouse was placed in a fear-conditioning chamber and was allowed to explore for 2 min, during which time freezing was recorded to assess the baseline freezing behaviour. Subsequently, a 30 s tone (70 dB) and light, ending with a 2 s foot shock (0.6 mA) were delivered. Then, 2 min later, a second unconditioned-stimulus–conditioned-stimulus pair was delivered. On the testing day (day 2), each mouse was first placed into the fear-conditioning chamber containing the same context, but with no CS or foot shock. Freezing was recorded for 2 min. Then, 1 h later, the mice were placed into a new context containing a different odour, cleaning solution, floor texture, chamber walls and shape. The animals could explore for 2 min before being re-exposed to the conditioned stimulus. Freezing was analysed for 1–3 min using a FreezeScan video tracking system and software (Cleversys).

### RAWM

Spatial learning and memory were assessed using the RAWM paradigm, according to an established protocol^58^. In this task, the mouse was trained to the location of a constant goal arm throughout the training and testing phase. The start arm changed each trial. Entry into an incorrect arm was scored as an error, and errors were averaged over training blocks (three consecutive trials). During training (day 1), the mice were trained for 12 trials (blocks 1–4), with trials alternating between a visible and hidden platform. After an hour break, learning was tested for 3 trials (block 5) using only a hidden platform. During testing (day 2), the mice were tested for 15 trials (blocks 6–10) with a hidden platform. When scoring, investigators were blinded to treatment.

### Data, statistical analyses and reproducibility

All experiments were randomized and blinded by an independent researcher before tail vein injection. Researchers remained blinded throughout histological, biochemical and behavioural assessments. Groups were unblinded at the end of each experiment on statistical analysis. Data are expressed as mean ± s.e.m. The distribution of data in each set of experiments was tested for normality using the D’Agostino–Pearson omnibus test or Shapiro-Wilk test. Statistical analysis was performed using Prism v.8.0 or v.9.0 (GraphPad). Means between two groups were compared using two-tailed unpaired Student’s *t*-tests. Comparisons of means from multiple groups with each other were analysed using one-way ANOVA followed by the appropriate post hoc test, as indicated in the figure legends. Trial by group interactions were analysed using repeated-measures ANOVA with Šidák’s correction for multiple comparisons. Additional statistical details are indicated in the respective figure legends. All data generated or analysed in this study are included in this Article. The main experimental findings are representative of two independently performed experiments. All replication attempts were successful. RNA-seq and CITE-seq data were not replicated due to resource limitations, but were orthogonally validated. Experimental replication was not attempted for negative data.

### Reporting summary

Further information on research design is available in the Nature Portfolio Reporting Summary linked to this article.

## Online content

Any methods, additional references, Nature Portfolio reporting summaries, source data, extended data, supplementary information, acknowledgements, peer review information; details of author contributions and competing interests; and statements of data and code availability are available at 10.1038/s41586-023-06436-3.

### Supplementary information

Supplementary Figure 1 Uncropped immunoblots for the Figures and Extended Data Figures.

Reporting Summary

Supplementary Table 1 RNA-seq analysis of hippocampi from aged mice treated with saline, young-mouse blood plasma preparations or the young-mouse platelet fraction. Spreadsheets of DEGs in the aged hippocampus relative to saline-treated controls after treatment with young-mouse blood plasma preparation or treatment with young-mouse platelet fraction, and the overlapping DEGs.

Supplementary Table 2 RNA-seq analysis of bulk microglia isolated from hippocampi of saline- or PF4-treated aged mice. Spreadsheet of DEGs in microglia isolated from the aged hippocampus after systemic administration of saline or PF4.

Supplementary Table 3 RNA-seq analysis of hippocampi from saline- or PF4-treated aged mice. Spreadsheet of DEGs in the aged hippocampus after systemic administration of saline or PF4.

Supplementary Table 4 RNA-seq analysis of hippocampi from saline or PF4 treated aged control and *Cxcr3*-KO mice. Spreadsheets of DEGs in the aged hippocampus of PF4 controls (WT) relative to saline, PF4 *Cxcr3*-KO mice relative to saline controls and PF4 controls, but not PF4 *Cxcr3*-KO mice, relative to saline controls.

### Source data

Source Data Fig. 1

Source Data Fig. 2

Source Data Fig. 3

Source Data Fig. 4

Source Data Fig. 5

Source Data Extended Data Fig. 1

Source Data Extended Data Fig. 2

Source Data Extended Data Fig. 3

Source Data Extended Data Fig. 5

Source Data Extended Data Fig. 6

Source Data Extended Data Fig. 7

Source Data Extended Data Fig. 8

## Figures and Tables

**Extended Data Table 1:**
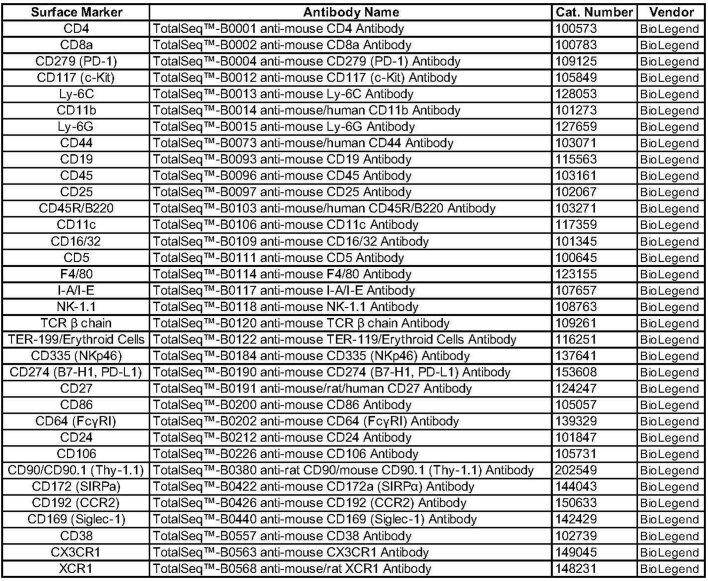
CITE-seq antibodies used for proteogenomic analysis of splenocytes from mice treated with saline or PF4

List of all surface markers, antibody names, catalogue number and vendor used for labelling splenocytes prior to CITE-seq library preparation.

## Data Availability

All data needed to understand and assess the conclusions of this study are included in the Article and the Supplementary Information. All bulk RNA-seq data supporting the findings of this study are available at the Gene Expression Omnibus (GEO) under accession number GSE173254, and the CITE-seq data are available at the GEO under accession number GSE179095. Human *CXCR3* expression data are available online (https://v21.proteinatlas.org/) and can be downloaded from http://www.proteinatlas.org/ENSG00000186810.tsv. Source data are provided with this paper.
