# Supplementary material for: Platelet factors attenuate inflammation and rescue cognition in ageing
Source: Nature. 2023 Aug 16;620(7976):1071–9. doi: 10.1038/s41586-023-06436-3 (PMC10468395; doi:10.1038/s41586-023-06436-3)
Supplement: Supplementary file 1 — Uncropped immunoblots for the Figures and Extended Data Figures. [file 41586_2023_6436_MOESM1_ESM.pdf]

nature portfolio

https://doi.org/10.1038/s41586-023-06436-3

Supplementary information

Platelet factors attenuate inflammation and rescue cognition in ageing

In the format provided by the authors and unedited

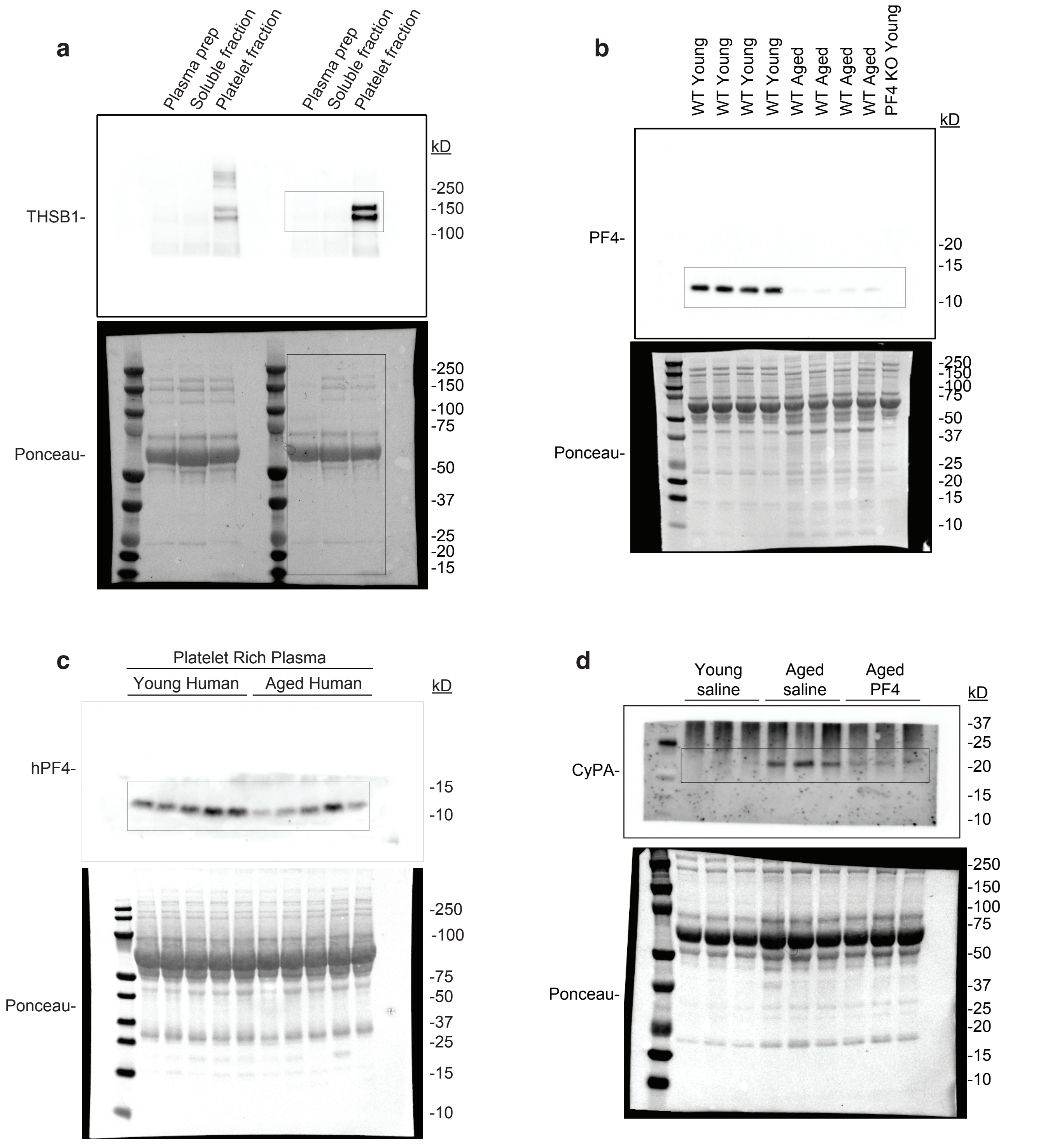

Supplementary Figure 1. Uncropped immunoblots from main and extended data figures. (**a**) Immunoblots for Figure 1d. Western blot of platelet marker Thrombospondin-1 (THSB-1) in the young plasma preparation, the platelet-depleted fraction, and the platelet fraction of mice, with an image of the gel stained with Ponceau. (**b**) Immunoblots for Figure 2a. Western blots of PF4 in the platelet fraction of plasma from young and aged mice, with an image of the gel stained with Ponceau. (**c**) Immunoblots for Figure 2c. Western blot of PF4 in platelet-rich plasma from young and aged humans, with an image of the gel stained with Ponceau. (**d**) Immunoblots for Extended Data Figure 2b. Western blot of Cyclophilin A (CyPA) in blood plasma preparation from young and aged saline treated and aged PF4 treated mice, with an image of the gel stained with Ponceau.
