# Supplementary material for: Platelet factors attenuate inflammation and rescue cognition in ageing
Source: Nature. 2023 Aug 16;620(7976):1071–9. doi: 10.1038/s41586-023-06436-3 (PMC10468395; doi:10.1038/s41586-023-06436-3)
Supplement: Supplementary file 2 — Reporting Summary [file 41586_2023_6436_MOESM2_ESM.pdf]

nature portfolio

Corresponding author(s): Saul Villeda and Adam Schroer

Last updated by author(s): 06/28/2023

Reporting Summary

Nature Portfolio wishes to improve the reproducibility of the work that we publish. This form provides structure for consistency and transparency in reporting. For further information on Nature Portfolio policies, see our Editorial Policies and the Editorial Policy Checklist.

Statistics

For all statistical analyses, confirm that the following items are present in the figure legend, table legend, main text, or Methods section.

n/a	Confirmed	
☐	☒	The exact sample size (*n*) for each experimental group/condition, given as a discrete number and unit of measurement
☐	☒	A statement on whether measurements were taken from distinct samples or whether the same sample was measured repeatedly
☐	☒	The statistical test(s) used AND whether they are one- or two-sided<br>*Only common tests should be described solely by name; describe more complex techniques in the Methods section.*
☐	☒	A description of all covariates tested
☐	☒	A description of any assumptions or corrections, such as tests of normality and adjustment for multiple comparisons
☐	☒	A full description of the statistical parameters including central tendency (e.g. means) or other basic estimates (e.g. regression coefficient) AND variation (e.g. standard deviation) or associated estimates of uncertainty (e.g. confidence intervals)
☐	☒	For null hypothesis testing, the test statistic (e.g. *F*, *t*, *r*) with confidence intervals, effect sizes, degrees of freedom and *P* value noted<br>*Give P values as exact values whenever suitable.*
☒	☐	For Bayesian analysis, information on the choice of priors and Markov chain Monte Carlo settings
☒	☐	For hierarchical and complex designs, identification of the appropriate level for tests and full reporting of outcomes
☒	☐	Estimates of effect sizes (e.g. Cohen's *d*, Pearson's *r*), indicating how they were calculated

Our web collection on statistics for biologists contains articles on many of the points above.

Software and code

Policy information about availability of computer code

Data collection	Illumina HiSEq 2500 (paired reads 2 × 100 bp); CFX384 Real Time System (Bio-Rad); Zeiss LSM800 confocal microscope; Zeiss LSM900 confocal microscope; ChemiDoc System (BioRad); Cytation 5 (BioTek); NovaSeq 6000 S2; Smart Video Tracking Software (Panlab; Harvard Apparatus); FreezeScan video tracking system (Cleversys, Inc); BD LSRII Flow Cytometer
Data analysis	Statistical analysis was performed with Prism 8.0 or 9.0 software (GraphPad Software).<br>ImageJ software (Version 2.0.0); FlowJo Version 10<br>For bulk RNA-sequencing alignment of sequencing reads to the mouse mm10 transcriptome was performed using STAR v2.7.3a39 following ENCODE standard options, read counts were generated using RSEM v1.3.1, and differential expression analysis was performed in R v3.6.1 using the DESeq2 package v1.38.040 (detailed pipeline v2.0.1 and options available on https://github.com/emc2cube/Bioinformatics/).<br>For CITE-sequencing the raw base sequence calls were demultiplexed into sample-specific cDNA and ADT files with bcl2fastq / mkfastq sample sheet through Cell Ranger (10X Genomics; version 5.0.1). CITE-seq analysis and statistical analysis of Raw FASTQ files were processed using the Cell Ranger software package (10X Genomics; version 5.0.2) for RNA expression matrix and CITE antibody counts matrix. The data were combined using a Cell Ranger aggrpipeline (10X Genomics). Downstream single cell analysis was performed using the R package Seurat (Version 4.0.0).<br>Gene ontology enrichment analysis was performed using Enrichr (GO Biological Process 2018; https://maayanlab.cloud/Enrichr/). Heatmaps were generated using Morpheus (https://software.broadinstitute.org/morpheus).

For manuscripts utilizing custom algorithms or software that are central to the research but not yet described in published literature, software must be made available to editors and reviewers. We strongly encourage code deposition in a community repository (e.g. GitHub). See the Nature Portfolio guidelines for submitting code & software for further information.

Data

Policy information about availability of data

All manuscripts must include a data availability statement. This statement should provide the following information, where applicable:

- Accession codes, unique identifiers, or web links for publicly available datasets
- A description of any restrictions on data availability
- For clinical datasets or third party data, please ensure that the statement adheres to our policy

All data needed to understand and assess the conclusions of this study are included in the text, figures, and supplementary materials. All bulk RNA-sequencing data that support the findings of this study are available in GEO with the accession number GSE173254, and the CITE-sequencing data are available in GEO with the accession number GSE179095. Human CXCR3 expression data are available at v21.proteinatlas.org and downloadable at http://www.proteinatlas.org/ENSG00000186810.tsv

Field-specific reporting

Please select the one below that is the best fit for your research. If you are not sure, read the appropriate sections before making your selection.

☒ Life sciences ☐ Behavioural & social sciences ☐ Ecological, evolutionary & environmental sciences

For a reference copy of the document with all sections, see nature.com/documents/nr-reporting-summary-flat.pdf

Life sciences study design

All studies must disclose on these points even when the disclosure is negative.

Sample size	The numbers of samples used were found to be sufficient to result in statistically significant differences using standard power calculations with alpha = 0.05 and a power of 0.8. We use an online tool (http://www.bu.edu/orccommittees/iacuc/policies-and-guidelines/sample-size-calculations/) to calculate power and sample size based on experience with the respective tests, variability of the assays, and inter-individual differences between groups.
Data exclusions	All exclusion criteria were pre-established. For novel object recognition, mice that did not explore both objects during the training phase were excluded from analysis. For Y maze, mice that did not perform three entries during the first minute of testing were excluded. For qPCR, sample was omitted due to inadvertent technical preparation issues.
Replication	The main experimental findings are representative of two independently performed experiments. All replication attempts were successful. RNA-seq and CITE-seq data were not replicated due to resource limitations, but were orthogonally validated. Experimental replication was not attempted for negative data.
Randomization	All experiments were randomized and blinded by an independent researcher before tail vein injection.
Blinding	Researchers remained blinded throughout histological, biochemical and behavioral assessments. Groups were un-blinded at the end of each experiment upon statistical analysis.

Reporting for specific materials, systems and methods

We require information from authors about some types of materials, experimental systems and methods used in many studies. Here, indicate whether each material, system or method listed is relevant to your study. If you are not sure if a list item applies to your research, read the appropriate section before selecting a response.

Materials & experimental systems

n/a	Involved in the study	
☐	☒	Antibodies
☒	☐	Eukaryotic cell lines
☒	☐	Palaeontology and archaeology
☐	☒	Animals and other organisms
☐	☒	Human research participants
☒	☐	Clinical data
☒	☐	Dual use research of concern

Methods

n/a	Involved in the study	
☒	☐	ChIP-seq
☐	☒	Flow cytometry
☒	☐	MRI-based neuroimaging

Antibodies

Antibodies used	For immunohistochemistry:<br>anti-Iba-1 (1:1000, Wako 0191741; or 1:1000, Synaptic Systems 234-004)

anti-CD68 [clone FA-11] (1:250, Bio-Rad MCA1957)
anti-C1q (1:500, Abcam ab182451, clone 4.8)
anti-phospho-CREB (Ser 133) (1:2500, Millipore 06-519)
donkey anti-rabbit conjugated Alexa Fluor® 555 (1:750, Life Technologies A31572)
donkey anti-rat conjugated Alexa Fluor® 647PLUS (1:750, Initrogen A48272)
donkey anti-guinea pig conjugated Alexa Fluor® 488 (1:750, Jackson ImmunoResearch 706-545-148)
goat anti-rabbit, biotinylated (1:500, Vector BA-1000)

For Western blot analysis:
anti-GAPDH [clone 6C5] (1:5000, Abcam, ab8245)
anti-mouse CXCL4/PF4 (1 µg/mL, R&D Systems, AF595)
anti-human CXCL4/PF4 [Clone 170138] (0.5 µg/mL, R&D Systems, MAB7952)
anti-Cyclophilin A (1:200, ENZO Life Sciences, BML-SA296-0100)
anti-Thrombospondin-1 [clone A6.1] (1:200, Santa Cruz, sc-59887, lot # C2519)
donkey anti-goat conjugated HRP (1:2000, Invitrogen, A15999)
goat anti-mouse conjugated HRP (1:2000, Millipore, AP124P)
donkey anti-rabbit conjugated HRP (1:2000, GE Healthcare, NA934V)

For ELISA:
PF4 (Mouse CXCL4/PF4 Quantikine ELISA Kit; R&D Systems, MCX400)
CCL2 (Mouse CCL2/JE/MCP-1 Quantikine ELISA Kit; R&D Systems, MJE00B),
TNFα (Mouse TNF-alpha Quantikine ELISA Kit; R&D Systems, MTA00B)
β2-Microglobulin (Cloud-Clone Corp, SEA260Mu)

For Flow Cytometry:
anti-CD61 PE [clone 2C9.G2 (HMβ3-1)] (1:50, BioLegend, 104308)
anti-CD45 BUV395 [Clone 30-F11] (1:200, BD, 564279)
anti-CD3 APC [clone 17A2] (1:200, Tonbo Biosciences, 20-0032-U025)
anti-B220 APC [clone RA3-6B2] (1:200, BioLegend, 103211)
anti-CD49b APC [clone DX5] (1:200, eBioscience, 50-112-9698)
anti-Ly6G BV711 [clone 1A8] (1:200, BioLegend, 127643)
anti-I-A/I-E Alexa Fluor® 700 [clone M5/114.15.2] (1:200, BioLegend, 107621)
anti-F4/80 PeCy7 [clone BM8] (1:200, eBioscience, 25480182)
anti-CD11b BV650 [clone M1/70] (1:200, BioLegend, 101239)
anti-CD45 BV711 [Clone 30-F11] (1:200, BD, 563709)
anti-B220 PeCy5 [clone RA3-6B2] (1:200, eBioscience, 15-0452-82)
anti-CD4 PeCy7 [clone RM4-5] (1:200, eBioscience, 25-0042-82)
anti-CD8a Pacific Blue™ [clone 5H10] (1:200, ThermoFisher, MCD0828)
anti-CD62L PerCP-Cyanine5.5 [clone MEL-14] (1:100, Tonbo Bioscience, 65-0621-U100)
anti-CD44 APC eFluor®780 [clone IM7] (1:100, eBioscience, 47-0441-82)
anti-CD3 eFluor® 660 [clone 17A2] (1:100, eBioscience, 50-0032-82)
anti-CD8a PE [clone 53-6.7] (1:100, BioLegend, 100708)
anti-CD279/PD-1 FITC [clone 29F.1A12] (1:200, BioLegend, 135214)

Validation

All antibodies are from commercially available sources and have been validated by the supplier for the indicated species and application utilized in our study. Manufacturers' websites contains validation and publications supporting the antibodies use for each species and assay employed.

Animals and other organisms

Policy information about studies involving animals; ARRIVE guidelines recommended for reporting animal research

Laboratory animals

All experiments were performed with mice on the C57BL/6 background. All studies performed with young and aged mice were performed with either 3 month-old or 20 month-old mice (The Jackson Laboratory and National Institutes of Aging). Homozygous Pf4 knockout mice (PF4 KO) were previously generated, characterized and provided as a generous gift from M. Anna Kowalska. Heterozygous mice were bred to generate PF4 KO and WT littermate controls that were used as mature adult (6-8 months) and middle-aged male mice (12-14 months). For Cxcr3 experiments, male CXCR3 KO and WT controls, and female CXCR3 KO and heterozygous control mice were used. All other studies were performed with male mice, except for validation of PF4's pro-cognitive effect in aged female mice. Mice were housed under specific pathogen-free conditions under a 12-hour light-dark cycle, with humidity maintained between 30-70% and temperature between 68-79 degrees F.

Wild animals

No wild animals were used in this study.

Field-collected samples

No field collected samples were used in this study.

Ethics oversight

All animal handling and use was in accordance with institutional guidelines approved by the University of California San Francisco IACUC.

Note that full information on the approval of the study protocol must also be provided in the manuscript.

Human research participants

Policy information about studies involving human research participants

Population characteristics	Samples were collected from healthy young men (20-35 years) or healthy older men (60-75 years).
Recruitment	Blood was collected from healthy young men (20-35 years) or healthy older men (60-75 years), who volunteered for either a cross-sectional or non-randomized single-arm study at the UCSF Human Performance Center. Participants were recruited from the San Francisco bay area, with primary recruitment at UCSF Health. There were no biases that impacted the study. Samples utilized in this experiment were from the cross-sectional baseline timepoint only.
Ethics oversight	This study was approved by the Institutional Review Board of UCSF

Note that full information on the approval of the study protocol must also be provided in the manuscript.

Flow Cytometry

Plots

Confirm that:

☒ The axis labels state the marker and fluorochrome used (e.g. CD4-FITC).

☒ The axis scales are clearly visible. Include numbers along axes only for bottom left plot of group (a 'group' is an analysis of identical markers).

☒ All plots are contour plots with outliers or pseudocolor plots.

☒ A numerical value for number of cells or percentage (with statistics) is provided.

Methodology

Sample preparation	Whole blood was collected via cardiopuncture and either diluted in anticoagulant or centrifuged to collect the platelet fraction of plasma.<br>For splenocyte isolation, spleens were removed, mechanically dissociated with a syringe plunger over a 70 µm cell strainer, and washed with 10 mL of ice-old RPMI media with 2% FBS. Cells were centrifuged and RBC lysis performed (155 mM NH_4Cl, 1 mM $KHCO_3$, and 0.1 mM EDTA). Subsequently, cells were washed and resuspended in staining buffer.
Instrument	BD LSR II Flow Cytometer
Software	FloJo v10
Cell population abundance	Sorted samples were >95% pure
Gating strategy	This information can be found in Fig. 1 and Extended Data Fig. 5. Briefly, for Figure 1 cells were gated by forward scatter and side scatter to identify objects the size of platelets. Of these cells, platelets were confirmed as CD61+ cells. For Extended data figure 5a-c, flow cytometry gating was used for the identification of CD45+ myeloid (lin-; i.e., CD3-, B220-, CD49b-) and lymphoid (lin+; i.e., CD3+, B220+, CD49b+) cells, neutrophils (CD45+, lin-, Ly6G+), and macrophages (CD45+, lin-, F4/80+, CD11b+) in the aged spleen. For Extended data figure 5e-f, flow cytometry gating was used for the identification of CD4+ T cells (CD45+, CD3+, CD4+), CD8+ T cells (CD45+, CD3+, CD8+), and CD8+ T effector memory cells (CD45+, CD3+, CD8+, CD44-HI, CD62L-LO) in the aged spleen. For Extended data figure 5h-i, flow cytometry gating was used for the identification of the exhaustion marker PD1 in CD4+ (CD3+, CD4+, PD1-HI) and CD8+ T cells (CD3+, CD8+, PD1-HI) following in vitro activation of aged T cells.

☒ Tick this box to confirm that a figure exemplifying the gating strategy is provided in the Supplementary Information.
